# Geoarchaeology reveals development of terrace farming in the Northern Apennines during the Medieval Climate Anomaly

**DOI:** 10.1038/s41598-025-08396-2

**Published:** 2025-07-10

**Authors:** Filippo Brandolini, Tim C. Kinnaird, Aayush Srivastava, Stefano Costanzo, Chiara Compostella, Sam Turner

**Affiliations:** 1 MIT Center for Sustainability Science and Strategy, Massachusetts Institute of Technology, 77 Massachusetts Avenue, Cambridge, 54-1312, 02139 MA United States; 2 School of Earth and Environmental Sciences, University of St Andrews, KY16 9TS St Andrews, Scotland; 3 Dipartimento di Scienze della Terra “Ardito Desio”, Università degli Studi di Milano, Milan, 20133 Italy; 4 Centre for Landscape - School of History, Classics and Archaeology, Newcastle University, Armstrong Building, Newcastle upon Tyne, Newcastle, NE17RU UK

**Keywords:** Anthropogenic Geomorphology, Land-Use change, Landscape Archaeology, Dantean Anomaly, Little Ice Age, Encastellation, OSL-PD, Geochronology, Isotopic analysis, Canossa, Environmental social sciences, Solid Earth sciences, Climate change

## Abstract

This study employs Optically Stimulated Luminescence Profiling and Dating (OSL-PD) to address the challenge of synchronizing social changes with natural events, a significant limitation in existing studies on the resilience and vulnerability of pre-modern societies to ecological stress. By uncovering the construction dates of terrace farming systems in the northern Apennines region, the research reveals a distinct temporal framework, indicating that the establishment of agricultural terraces predominantly occurred during the 11th to 13th centuries CE. This crucial time frame aligns directly with complex socio-economic factors, including the encastellation process, alongside the climatic shifts characterising the Medieval Climate Anomaly. Isotopic fractionation of the Total Organic Carbon confirms that different agricultural choices were made in coincidence with the establishment of terrace farming. The resultant historical rural landscape underwent continuous enhancements in the centuries that followed. Notably, the main phases of (re)construction correspond to the coldest periods of the Little Ice Age, offering new insights into the historical interactions between human activities and the environment during the Late Holocene in the area.

**Supplementary Information:**

The online version contains supplementary material available at 10.1038/s41598-025-08396-2.

## Introduction

Understanding the intricate connections between ecosystems, social organisation, human actions, and the potential for adaptation to climate change requires research that spans diverse fields of knowledge^1^. Information from archaeology concerning historical climates and environments serves as a valuable resource, offering insights into the challenges humans may encounter and the eventual effects of various short-term adaptive methods^2^. Archaeology can play an important role in addressing this global crisis by presenting evidence of how climate changed in specific regions over time, thereby affecting entire ecosystems^3,4^. Specifically, landscape archaeology^5^ and geoarchaeology^6^ offer a long-term perspective on how humans interacted with the environment and coped with challenging climatic variations, shaping many cultural landscapes through pre-industrial agricultural practices that trace their origins far back in history^7,8^. Until the mid-20th century CE, the historic rural landscape of the northern Apennines (Italy) was characterised by the presence of two enduring traditional farming methods: agroforestry systems^9^ and terrace farming^10^. However, starting from the 1950 s, the introduction of mechanisation in agriculture, coupled with new socio-economic needs, gradually triggered depopulation in the mountainous regions, fostering urbanisation in the floodplain areas, the shift from polyculture systems to monoculture, and the abandonment of terrace agriculture^11–14^. Agricultural terraces have been utilised for centuries across various parts of the world, having significant potential to mitigate environmental hazards including aspects of climate change^15^. Specifically, agricultural terraces serve to prevent soil erosion^11,16,17^ enhance soil water retention^18^ and contribute to carbon sequestration^19^. However, the origins of these distinctive landscape features have often proven elusive to researchers^20^. Establishing a connection between the inception of terrace farming and a specific Holocene climate phase can offer potential insights to better understand historical adaptations to climate fluctuations and ecological stress in the past^2^. Although excavations occasionally yield datable artefacts^21^disturbances like bioturbation (e.g. rooting) often complicate their interpretation, and artefacts are frequently altogether absent. Radiocarbon dating has been applied to buried soils in terrace studies, with statistical calibrations enhancing precision. However, various factors including erosion and redeposition and the presence of older carbon fractions in the environment can lead to misleading age estimates^22^. Luminescence dating, which reveals when minerals were last exposed to sunlight or heat, has also been employed to date terrace soils and construction layers^23^. However, conventional radiocarbon and luminescence methods only date the specific location sampled in the sedimentary profile, limiting their broader applicability. Recently, Optically Stimulated Luminescence Profiling and Dating (OSL-PD) has emerged as a valuable geoarchaeological technique for dating landscape features, providing insights into their genesis, evolution and eventual abandonment^24–26^. The OSL-PD method uses real-time luminescence profiling coupled with sediment analysis to construct on-site stratigraphies and permit focused sampling for OSL dating. This approach moves beyond isolated samples by creating a continuous profile through terrace sediments and thereby providing insights into their creation and change over time^24^. The technique has already been tested across the Mediterranean region, revealing that while many terraces were created in the first millennium BCE, some of the most intensive episodes of terrace building occurred between 1100 and 1600 CE^25,27,28^.

The origins and development of terrace farming in Italy remain somewhat unclear. Sporadic evidence suggests that terrace farming was already in use during the Neolithic era, but it seems to have become widespread only in the Middle Ages (ca. 5th to 14th century CE). During this period, agricultural terraces became an essential part of Italian landscapes, reaching their peak in the Renaissance (14th − 15th centuries)^29,30^. Archaeological studies in the northern Apennines have extensively explored the medieval origins of several historic settlements, particularly those associated with the encastellation process under Canossa rulers. However, the origin of the rural landscape heritage of the area remains still poorly investigated (see ‘Material and Methods’ section).

In this research, we employed OSL-PD in the northern Apennines (Italy) on historic terraced systems to gain insights into their chronological development. The results allow us to consider how the establishment, evolution, and abandonment of terraces may relate to Late Holocene climate fluctuations, as well as local historical and socio-cultural dynamics. Furthermore, isotopic fractionation of stable carbon isotopes (δ^13^C and δ^12^C) from the Total Organic Carbon (TOC) of the terrace sediment profiles has been employed. By analysing the differences in carbon isotope ratios (δ^13^C), various sources of organic carbon can be inferred, allowing for the assessment of contributions from different vegetation types and therefore different land use practices over time^31,32^. This analysis has been integrated in the research protocol, to uncover potential human-driven land-use strategies associated with the development and evolution of terrace farming in the region. Additionally, the analysis of TOC content and δ^13^C served as a validation test to investigate whether the OSL signal intensities in the area varied independently of bulk sediment properties (e.g. colour, organic content, clast content) across the sediment profiles.

In summary, this research aims to address the challenge of aligning social changes with climatic fluctuations in the northern Apennines by exploring:

1. the chronology of terrace farming establishment in the area;
2. phases of renovation and abandonment, correlating these with major Late Holocene climate phases, as well as local historical events and political factors that may have influenced the adoption of terrace farming practices in the region.

### Study area

**Fig. 1:**
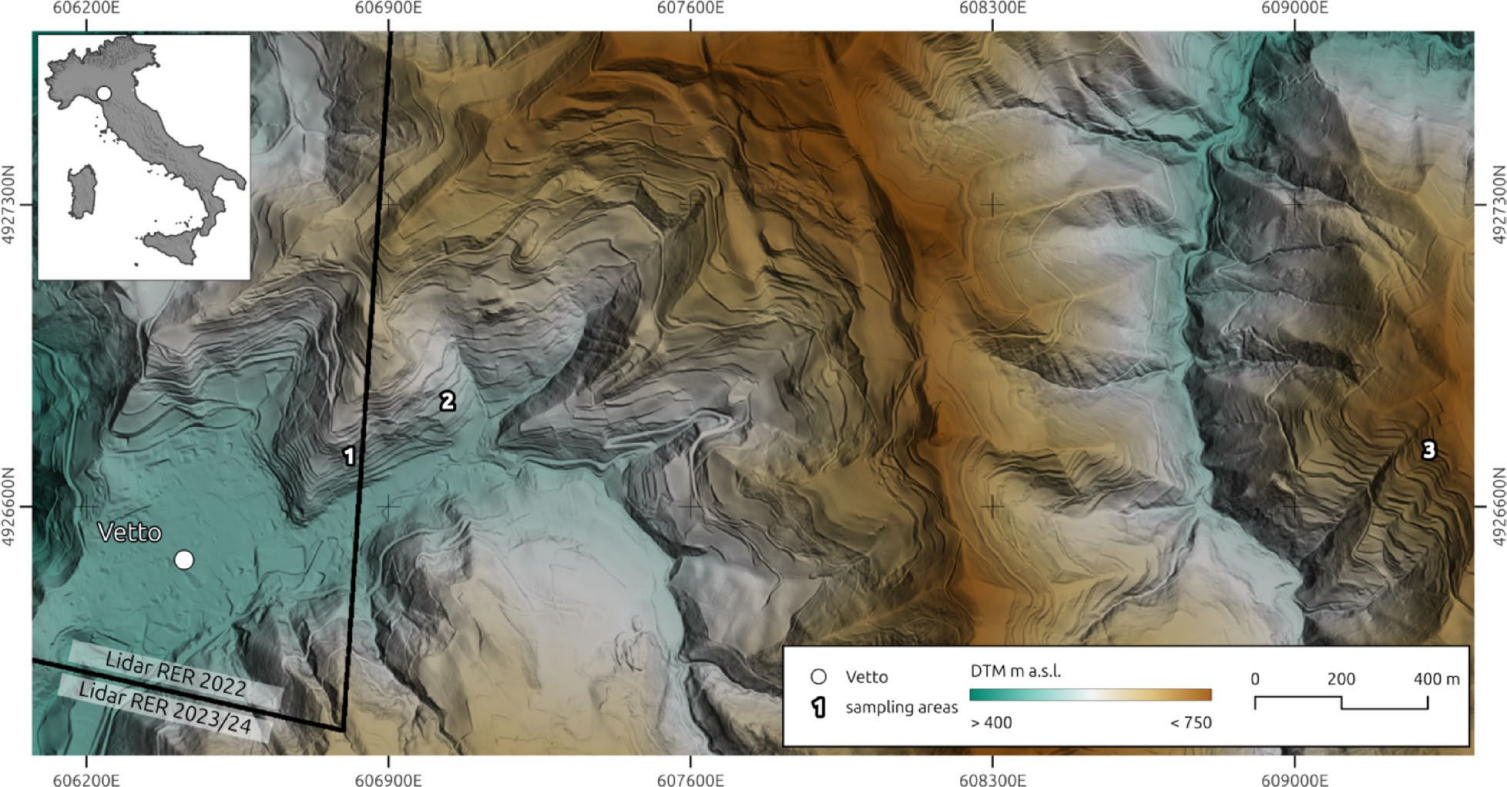
Location of the study area in the northern Apennines (Italy). Bold numbers indicate the sampling areas: 1 – Ferro; 2 – Castello; 3 – Pineto. The digital terrain model (DTM) was generated using two regional LiDAR datasets (RER 2022 and RER 2023/24) freely available from the Geoportale Emilia-Romagna^36^. Image generated with the software QGIS 3.42 (https://www.qgis.org/en/site/index.html*).*

**Fig. 2:**
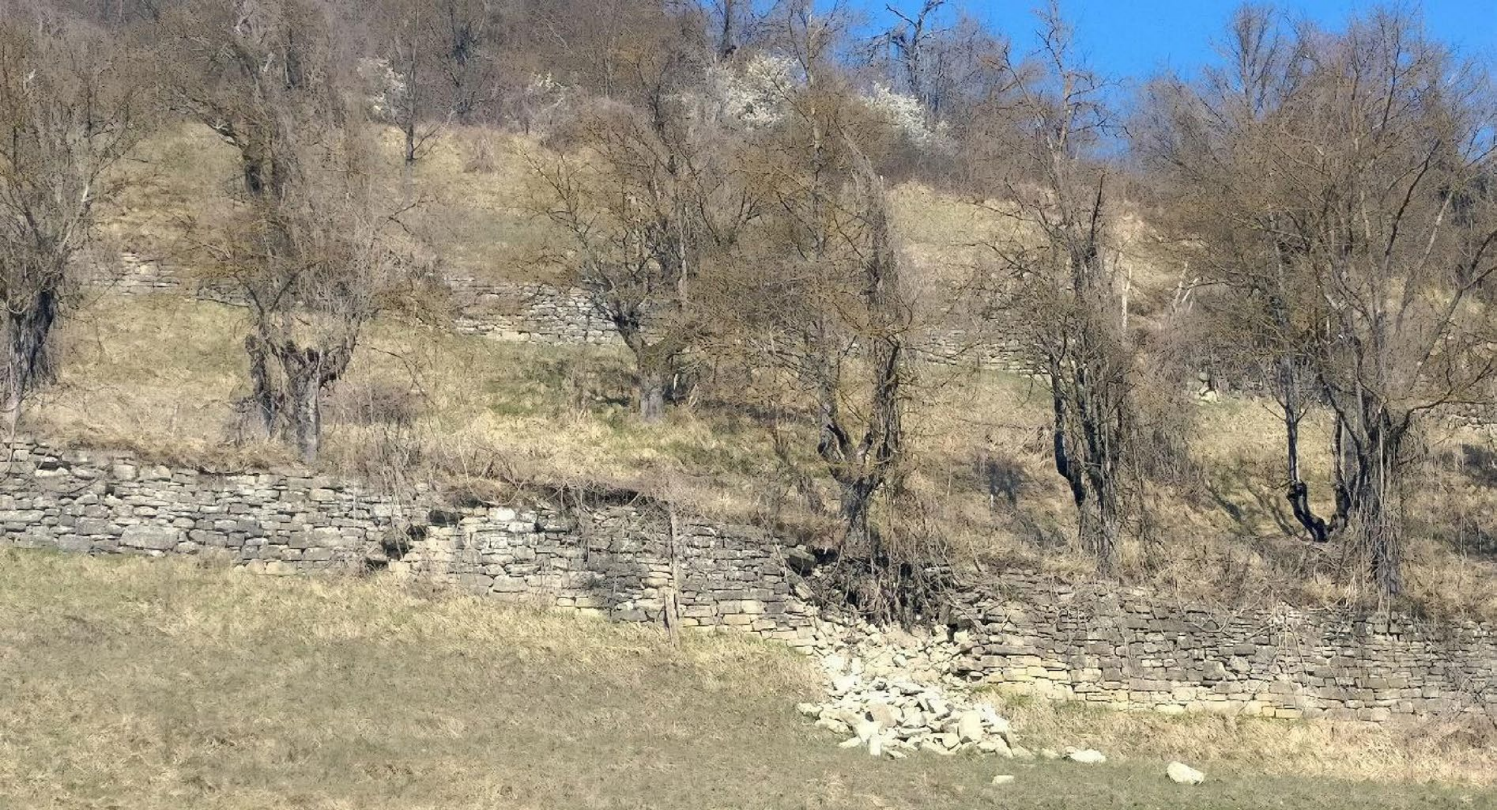
An example of historic terraced agroforestry systems in the study area.

This study focuses on a segment of the northern Apennines, specifically the municipality of Vetto d’Enza (commonly known as *Vetto*). Vetto is situated on the right bank of the Enza River within the Man and the Biosphere UNESCO reserve^33^ located in the Emilia-Romagna region of northern Italy (Fig. 1). The main defining features of the area’s landscape character are extensive stone walls and earth banks that were historically used to delineate tenurial boundaries and support agricultural terracing across steeply-sloping fields^14^ (Fig. 2). Vetto represents an ideal case study for exploring the link between past natural events and human resilience in the northern Apennines due to its well-preserved historic terrace systems, recently recognized by the Fondo Ambiente Italiano (FAI), the Italian National Trust responsible for preserving and conserving the national invaluable heritage^34^. These terraced systems were established on south and southwest-facing slopes adjacent to the town’s historic nucleus, known as *Castello*. They span elevations ranging from 450 to 570 m above sea level (Fig. 1). Despite their cultural significance, the origin of these terraced systems remains unresolved due to limited historical documentation. Historical accounts^35^ commonly attribute the construction of the dry-stone walls forming the terraced systems in Vetto to the late 18th and early 19th centuries^34^.

**Fig. 3:**
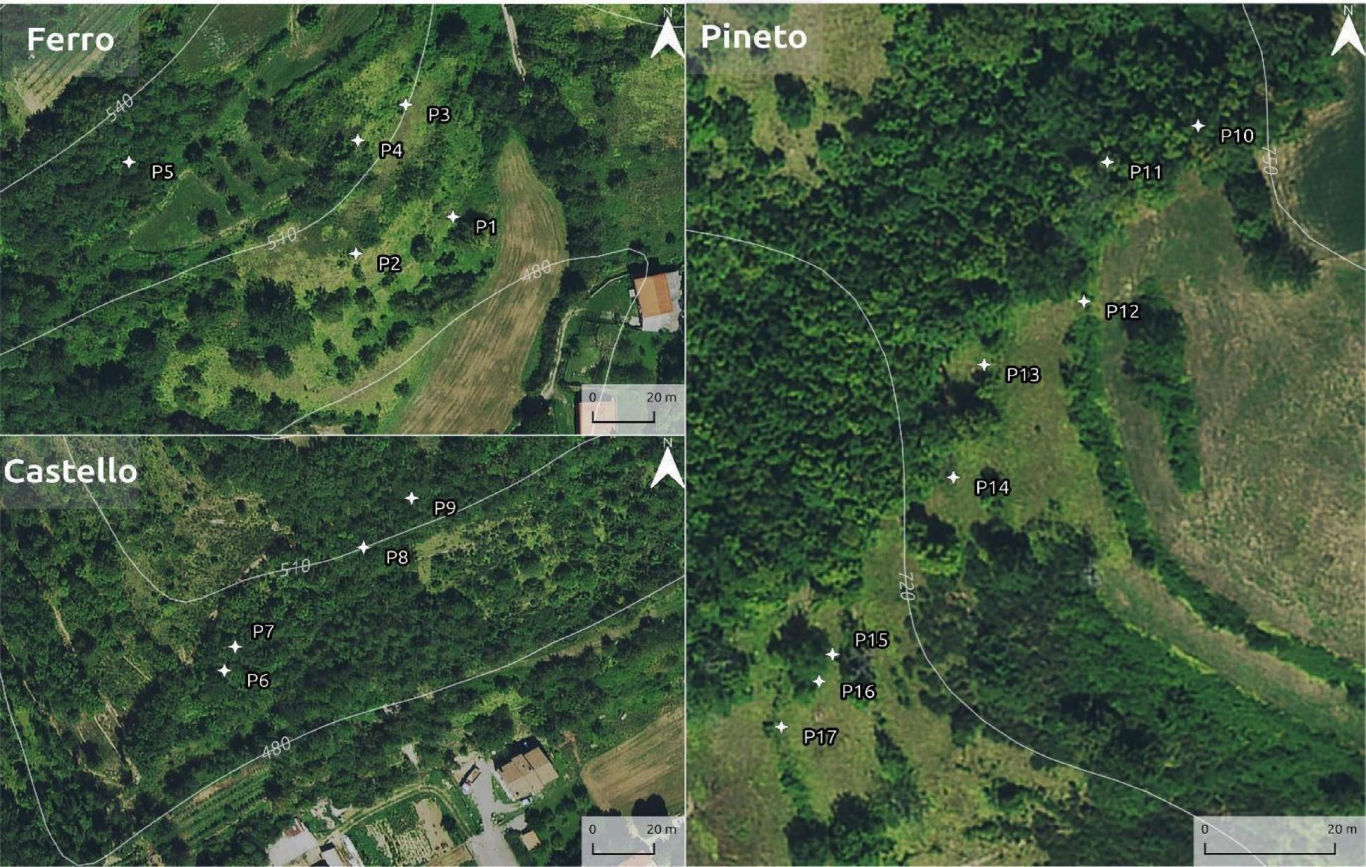
Location of the profiles sampled: P1 - P5 (Ferro); P6 - P9 (Castello), P10 - P17 (Pineto). Image generated with the software QGIS 3.42 (https://www.qgis.org/en/site/index.html*).*

Recently, preliminary OSL-PD results^17^ from the Vetto area, specifically the locality known as *Ferro*, attributed a general chronology to these landscape features, dating their initial establishment to the Early Middle Ages (5th – 10th centuries CE), with further construction phases occurring between the High Middle Ages (11th − 14th centuries CE) and the Modern Age (16th − 19th centuries CE). In this research, we build upon the OSL-PD data provided by Brandolini et al. (2023)^17^ augmenting it with new measurements from historic terraced systems in the northern Apennines. This allows for a more comprehensive understanding of their chronological and spatial development. In the present study, OSL-PD was applied to adjacent areas of *Castello* and *Pineto* to investigate whether the chronology of terraces identified in *Ferro* could be expanded, thereby providing a more robust interpretation of the historic landscape evolution in this portion of the northern Apennines (Fig. 3). Further details concerning the methodology are provided in the ‘Materials and Methods’ section.

## Results

A total of 250 samples were collected and screened with a SUERC portable OSL reader^37^ from across the terraced slopes of Ferro, Castello and Pineto (Figs. 1 and 3). Temporal control was provided from the chronology of terrace construction at Ferro^17^ augmented with additional constraints at Pineto (Table 1).

**Table 1:** Luminescence ages for Ferro and Pineto: burial doses, dose rates, and ages are shown to two decimal places, with all calculations made prior to rounding. All dates are relative to the year 2022. (modified from Brandolini et al. 2023^17^).

	Field code	Lab code/CERSA#	Depth/cm	Aliquots	Over-dispersion/%	Burial dose /Gy	Dose rate /Gy ka-1	Age/ka	Calendar years
Ferro	P2 OSL1	878	78	34 (36)	96 ± 12	0.66 ± 0.11	2.39 ± 0.12	0.28 ± 0.05	CE 1750 ± 50
	P2 OSL2	879	86	33 (36)	80 ± 10	0.94 ± 0.13	2.51 ± 0.12	0.37 ± 0.06	CE 1650 ± 60
	P3 OSL1	880	55	23 (36)	36 ± 6	2.34 ± 0.29	2.35 ± 0.11	0.99 ± 0.13	CE 1030 ± 130
	P4 OSL1	881	62	20 (24)	39 ± 6	1.41 ± 0.13	2.50 ± 0.12	0.57 ± 0.06	CE 1460 ± 60
	P4 OSL2	882	79	20 (24)	87 ± 15	1.63 ± 0.36	2.41 ± 0.12	0.68 ± 0.15	CE 1350 ± 150
	P5 OSL1	883	63	28 (44)	85 ± 11	2.80 ± 0.46	2.40 ± 0.11	1.17 ± 0.20	CE 860 ± 200
Pineto	P10 OSL1	889	81	24 (40)	47 ± 7	4.11 ± 0.67	2.56 ± 0.12	1.60 ± 0.27	CE 420 ± 270
	P11 OSL1	890	150	33 (42)	52 ± 6	1.88 ± 0.25	2.52 ± 0.12	0.75 ± 0.11	CE 1280 ± 110

Infra-Red Stimulated Luminescence (IRSL) net signal intensities ranged over three orders of magnitude, from 10^3^ to 10^6^ counts; OSL signal intensities, over a similar range, from 10^4^ to 10^7^ counts. Signal intensities are parameterised here, as elsewhere, as the total number of photon counts over 60 s, minus the background count^37,38^. The interpretation of OSL signal intensities, their depletion indices and the IRSL: OSL ratio have been discussed in a number of recent publications^24–26^. This attests to a long chronology to the sediments, and by association, the terrace walls (even withstanding variations in environmental dose rate). The relative luminescence stratigraphies at Ferro are discussed first, before expanding the discussion to Castello and Pineto. The luminescence intensities for each profile are available attached to this paper as Supplementary Materials (Table S1).

### Relative sediment chronologies

**Fig. 4:**
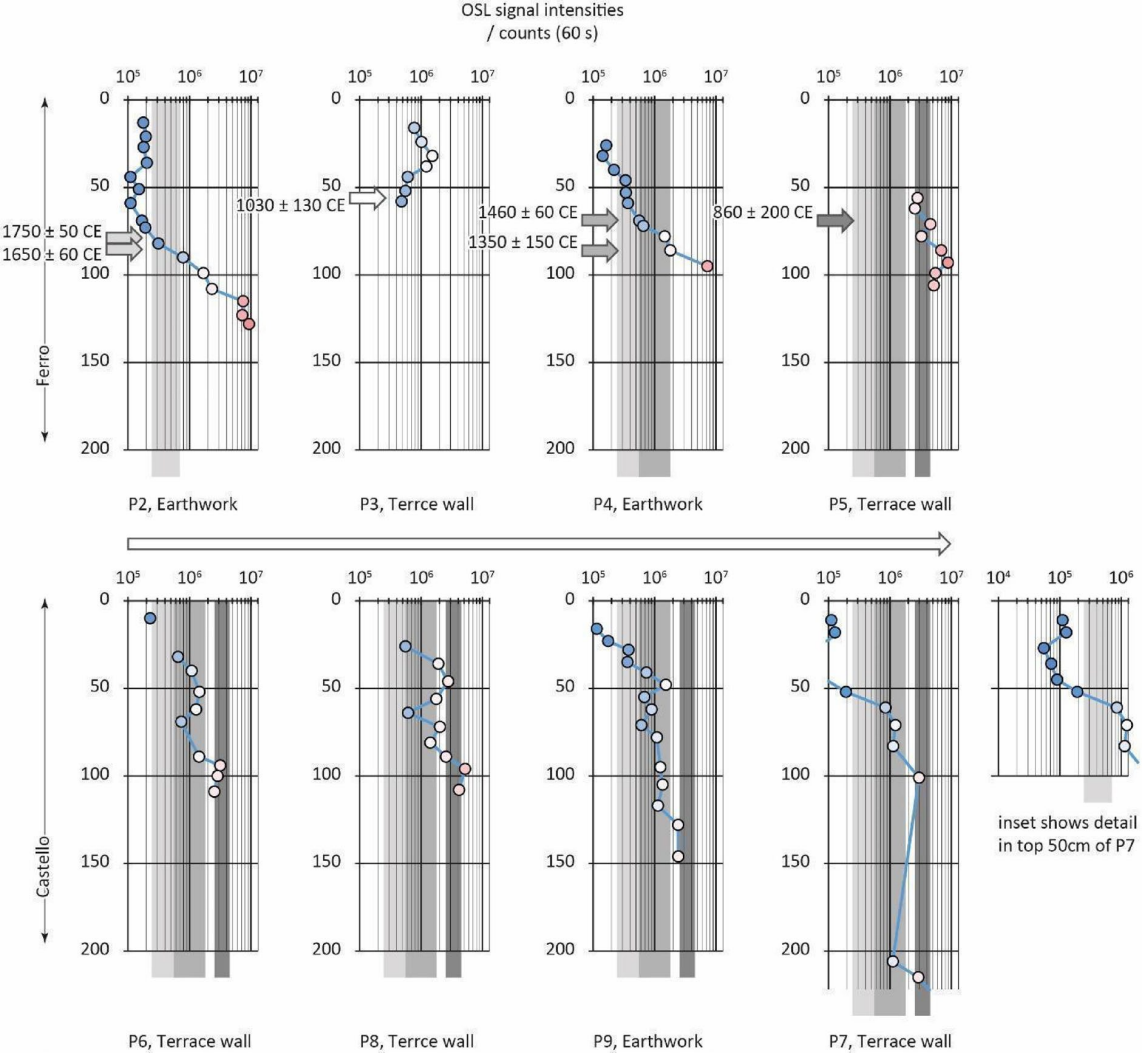
Luminescence stratigraphies for the soil-sediment profiles associated with terracing at Vetto: (top) intensity-depth profiles for the slope of Ferro, P2-P5, annotated with the quartz SAR OSL ages reported in Brandolini et al. (2023). The sampling spots are coloured to emphasis the range in intensities: the cooler colours indicate the low intensities; the warmer colours the higher intensities (the same colour scale is used in Supplementary Materials - Table S1).These ages are used to define discrete packages of sediment that are likely to have been deposited in the mid-17th to 18th centuries CE (light grey), the mid-14th to 15th centuries CE (grey), and pre-10th century CE (dark grey); (bottom) intensity-depth profiles for the slope of Castello, P6-P9. The magnitude and range in OSL intensities are similar to those observed at Ferro, allowing a first-order approximation of age for the sediments at Castello.

At Ferro, the sediments forming earthwork P2 (Figs. 3 and 4) show a normal-signal depth progression, from 1.76 × 10^5^ counts near the surface to 9.27 × 10^6^ counts at depth, with prominent step-changes across unit boundaries at 82–90 cm and 108–110 cm. The lower part of the profile (between 115 and 128 cm) is a compact gritty loam largely devoid of organic matter, forming the substrate on the slope, characterised by intensities greater than 7.45 × 10^6^ counts. Above this is a loam with some modest stratification, characterised by intensities from 2.31 × 10^6^ counts at the base, 108 cm deep, to 7.83 × 10^5^ counts at 90 cm deep; then a looser loam, with more abundant organic matter, characterised by intensities from 3.11 to 1.76 × 10^5^ counts, near the surface. The field interpretation was that the substrate was cleared or prepared to form a level surface for the construction of the earthwork, and dating samples were positioned accordingly, on either side of the unit boundary at 82–90 cm depth. These are the samples dated to 1750 ± 50 CE and 1650 ± 60 CE (Table 1).

**Fig. 5:**
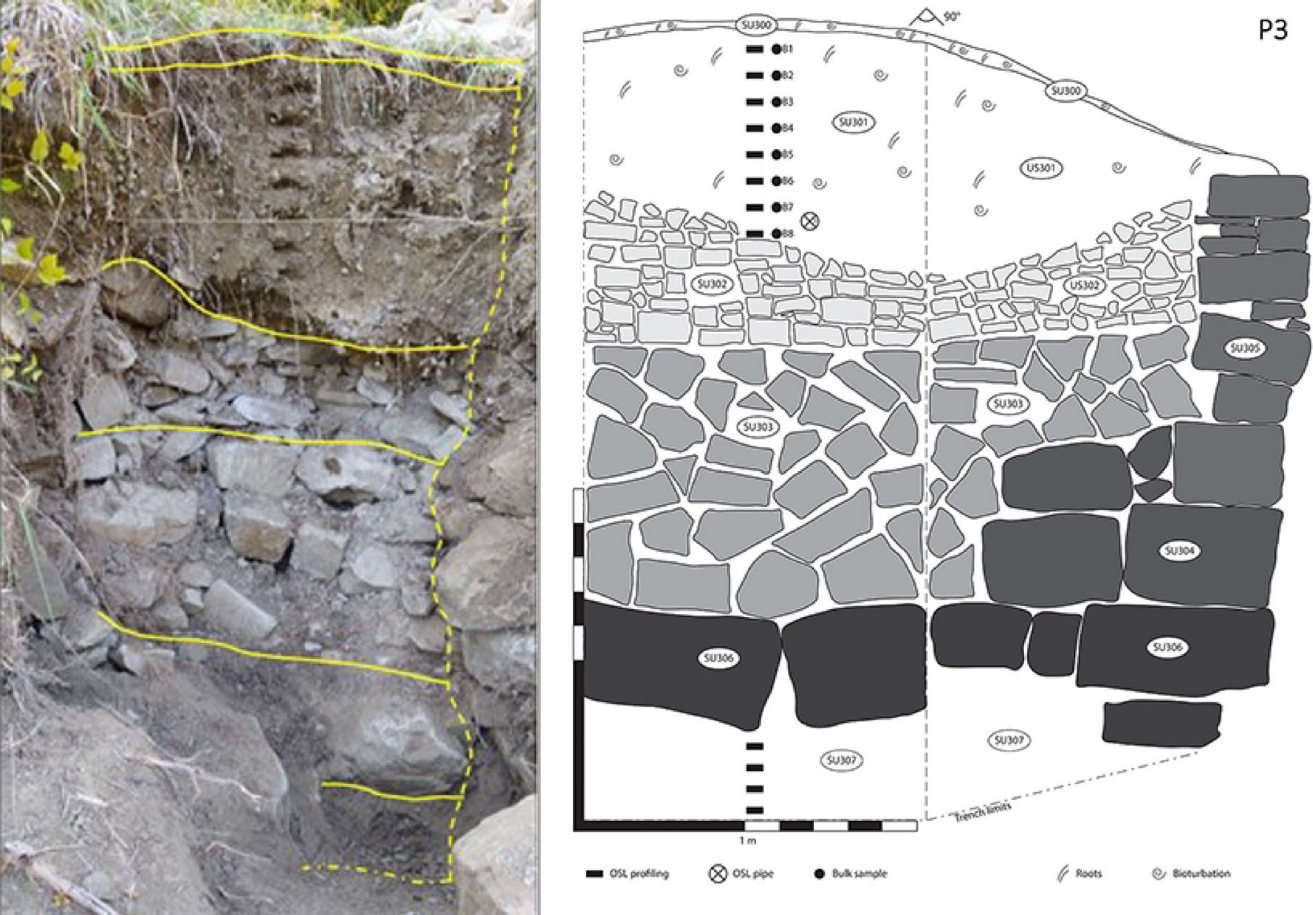
Stratigraphic section of profile number 3 (P3, Ferro): SU 300: Active organic litter, rich in gastropod shells and decaying plant matter; SU 301: loamy deposit, cleared of large stones and pebbles, constitutes the cultivable terrace fill. Modest humification and bioturbation, occasional presence of gastropod shells. Level surface extends for a few metres behind the excavated trench; SU 302 and 303: small to medium sized stones accumulated behind the outer walls for reinforcement, arranged into two structural elements separated by a planar interface; discontinuous presence of interstitial loam; SU 304 and 305: cuboid and oblate stones constituting the outer wall; size decreases with height; SU 306: wall basement, very large cuboid stones set underneath SU 304 and 303; hind limit not reached; SU 307: loamy regolith, compact and homogeneous. In all the profiles, the soil beneath the active organic litter is characterised by a compact, homogeneous mass with no marked stratification and an average pale yellowish-grey colour inherited from the bedrock and regolith (Munsell colour chart: 2.5Y 7/2, determined on ground dried bulks). Stratigraphic organisation of the profiles is mostly of pedogenetic nature, with the upper portions characterised by modest humification and bioturbation (i.e. presence of roots, channel voids and gastropod shells), while the lower portions display illuviation of calcium carbonate that accumulates forming gravel-sized nodules and contributing to the compaction of the otherwise brittle loam (Fig. 7). The OSL sample indicated in this figure corresponds to CERSA880 (Table 1).

The earthwork at P2 forms the lower unit to a terraced field which is bounded upslope by a terrace wall, sampled at P3 (Figs. 3 and 5). The substrate here, from 196 cm depth in profile, is characterised by intensities > 1.26 × 10^6^ counts. If the lower units in P2 and P3 are age equivalent, then this supports the hypothesis that the slope was levelled ahead of construction of the earthwork, with disturbance to and some bleaching of this sediment. The remainder of the profile was taken behind the terrace wall. At the base, above stones forming a backfill to the foundations of the wall, the loamy soils are characterised by intensities at ~ 5.0 × 10^5^ counts. This unit is dated to 1030 ± 130 CE (Table 1). Above this, the transition into the more organic, active soils is characterised by more mixed signals, > 1.22 × 10^6^ counts.

Above P3, the terraced field is bounded, upslope by an earthwork, P4 (Fig. 3). These sediments exhibit a similar profile to that observed for the earthwork below, progressing from 1.63 × 10^5^ counts near surface to 1.80 × 10^6^ counts at depth, increasing to > 7.2 × 10^6^ counts in the substrate. In terms of relative age though, these intensities are marginally larger than those observed at P2: this landscape feature should have been constructed earlier, and the depositional ages – 1460 ± 60 CE and 1350 ± 150 CE imply so (Table 1). Above P4 is a terrace wall, P5, that retains the cultivation soil, then a loam with some internal stratification, before encountering the substrate at depth. This profile is more complex, and although it does show an overall signal-depth progression, from 2.80 × 10^6^ counts, near surface to > 5.17 × 10^6^ counts at depth, there are deviations from this down-profile, with maxima > 8.75 × 10^6^, attesting to episodic colluviation. Notwithstanding this, these sediments are characterised by the highest intensities observed on the Ferro slope: this feature should be the oldest preserved here, and the depositional age, 860 ± 200 CE confirms this (Table 1).

A relative time-depth can be established for Ferro: > 3.4 × 10^6^ counts, likely earlier than mid-9th century CE; < 3.1 × 10^5^ counts (dark grey; Fig, 3), likely later than the 17th century CE (light grey). This is supported by the soil geochemistry; trace element concentrations were determined by inductively coupled plasma mass spectrometry (ICP-MS; U, Th) and major elements by inductively coupled plasma optical emission spectrometry (ICP-OES; K) at X-Ray Mineral Services, UK (Table 2). The sediments at Ferro, irrespective of whether they constitute an earthwork or are retained behind a terrace wall, show little spatial variation in radionuclide concentration. This is expected: the sediment stratigraphies associated with the terrace walls and earthworks are similar, with the substrate developed on the weathered C horizon, then a compact silt loam, transiting into the cultivation and active soils (see ‘Material and Methods’).

The terraced slope at Castello is located 200–300 m SW of Ferro (Figs. 1 and 3). The first terrace investigated in the sequence, P6, was only partly sectioned, as after cursory cleaning a stone wall was observed to survive at depth. The profile examines the cultivation soil, 0 to ca. 35 cm, then the underlying loam, ca. 35 to ca. 65 cm, before encountering constructional rubble through ca. 65 to ca. 100 cm. The lowest sample in the sequence is within the clay substrate. Through the cultivation soil, OSL signal intensities increase from 2.29 to 6.51 × 10^5^ counts; beneath this, the loam is characterised by intensities in the range 1.1–1.4 × 10^6^ counts (Fig. 4). It is not possible to distinguish, in time-depth, between the rubble fill, 2.86 to 3.15 × 10^6^ counts, and the clay substrate, 2.53 × 10^6^ counts. In terms of the relative time-depth established at Ferro, the cultivation soils are likely contemporary with the mid-17th −18th centuries CE soils identified in earthwork P2, with the terrace wall built (or rebuilt) earlier, synchronous with the construction of earthwork P4 in the mid-14th century CE (Fig. 4).

**Table 2:** Radionuclide concentrations by ICP-OES (K) and ICP-MS (U, Th). All values are shown to two decimal places, all calculations made prior to rounding. (modified from Brandolini et al. 2023^17^).

Lab code	ICP-OES and ICP-MS			Ḋβ/Gy.ka^−1^	Ḋγ/Gy.ka^−1^	Ḋcosmic/Gy.ka^−1^	Total Ḋ/Gy.ka^−1^
	Th/ppm	U/ppm	K/%				
878	8.4 ± 0.8	2.2 ± 0.2	1.59 ± 0.16	1.34 ± 0.10	0.86 ± 0.05	0.19 ± 0.02	2.39 ± 0.12
879	8.6 ± 0.9	2.2 ± 0.2	1.58 ± 0.16	1.42 ± 0.11	0.91 ± 0.05	0.19 ± 0.02	2.51 ± 0.12
880	8.7 ± 0.9	2.4 ± 0.2	1.59 ± 0.16	1.30 ± 0.10	0.84 ± 0.05	0.21 ± 0.02	2.35 ± 0.11
881	8.8 ± 0.9	2.4 ± 0.2	1.61 ± 0.16	1.40 ± 0.11	0.90 ± 0.05	0.20 ± 0.02	2.50 ± 0.12
882	9.0 ± 0. 9	2.42 ± 0.2	1.61 ± 0.16	1.34 ± 0.10	0.88 ± 0.05	0.19 ± 0.02	2.41 ± 0.12
883	9.0 ± 0. 9	2.4 ± 0.3	1.54 ± 0.15	1.32 ± 0.10	0.88 ± 0.05	0.20 ± 0.02	2.40 ± 0.11
889	9.2 ± 0.9	2.1 ± 0.2	1.54 ± 0.15	1.43 ± 0.11	0.94 ± 0.06	0.20 ± 0.02	2.56 ± 0.12
890	8.7 ± 0.9	2.1 ± 0.2	1.46 ± 0.15	1.41 ± 0.11	0.93 ± 0.06	0.18 ± 0.02	2.52 ± 0.12

Further up the slope, a small terrace containing a shepherd’s hut is bounded by terrace walls P9 (downslope) and P8 (upslope) (Fig. 3). Again, it was not possible to fully excavate P9 or P8, as the base of both exploratory trenches encountered large stone boulders and other rubble. The profile through P9 shows a signal-depth progression through 1.14 × 10^5^ counts to 2.42 × 10^6^ counts, with prominent step changes across 35–41 cm depth, 71–78 cm depth and 117–128 cm depth. The first coincides with the transition from cultivation soil to more compact silt loam, with some internal stratification; the second with the base of a rubble horizon, attributed to construction of the wall. A similar profile is observed in P8, although the top part of this is more disturbed, with deviations from the signal-depth progression through 36 to 64 cm, suggesting an increased colluvial component to the soil. Beneath though, the profile shows a more straightforward progression from 6.12 × 10^5^ counts to > 2.54 × 10^6^ counts, with boundaries at 81–89 cm depth and 89–96 cm depth. As at P6, these sequences can be placed within the relative time-depth established at Ferro, suggesting deposition – potentially associated with establishment (or restoration) of the terrace walls - in the mid-14th century CE (Fig. 4).

The highest terrace wall on the slope, P7, was partly collapsed, permitting access to the sediments in two parts: the top of the profile examines the cultivation soils retained behind and above the prominent wall, and the bottom part examines the loams that have partly infiltrated the foundation fills. The soils show a progression from 5.44 × 10^4^ counts (excluding the top, recently re-deposited materials) to 1.11 × 10^6^ counts, with prominent step changes between 45 and 53 cm depth (base of topsoil) and 52–61 cm depth (transition to a gravellier unit). Beneath the wall, the loams show a progression from 1.11 to > 6.50 × 10^6^ counts, with some local deviations attesting to re-deposited materials, poorly reset, and potentially associated with construction. In relative time-depth, the cultivation soils are likely young, more recent than the late 18th century CE; the lower loams, the mid-14th −15th centuries CE; with construction likely synchronous with the upper terrace at Ferro, in the 9th century CE (Fig. 4).

**Fig. 6:**
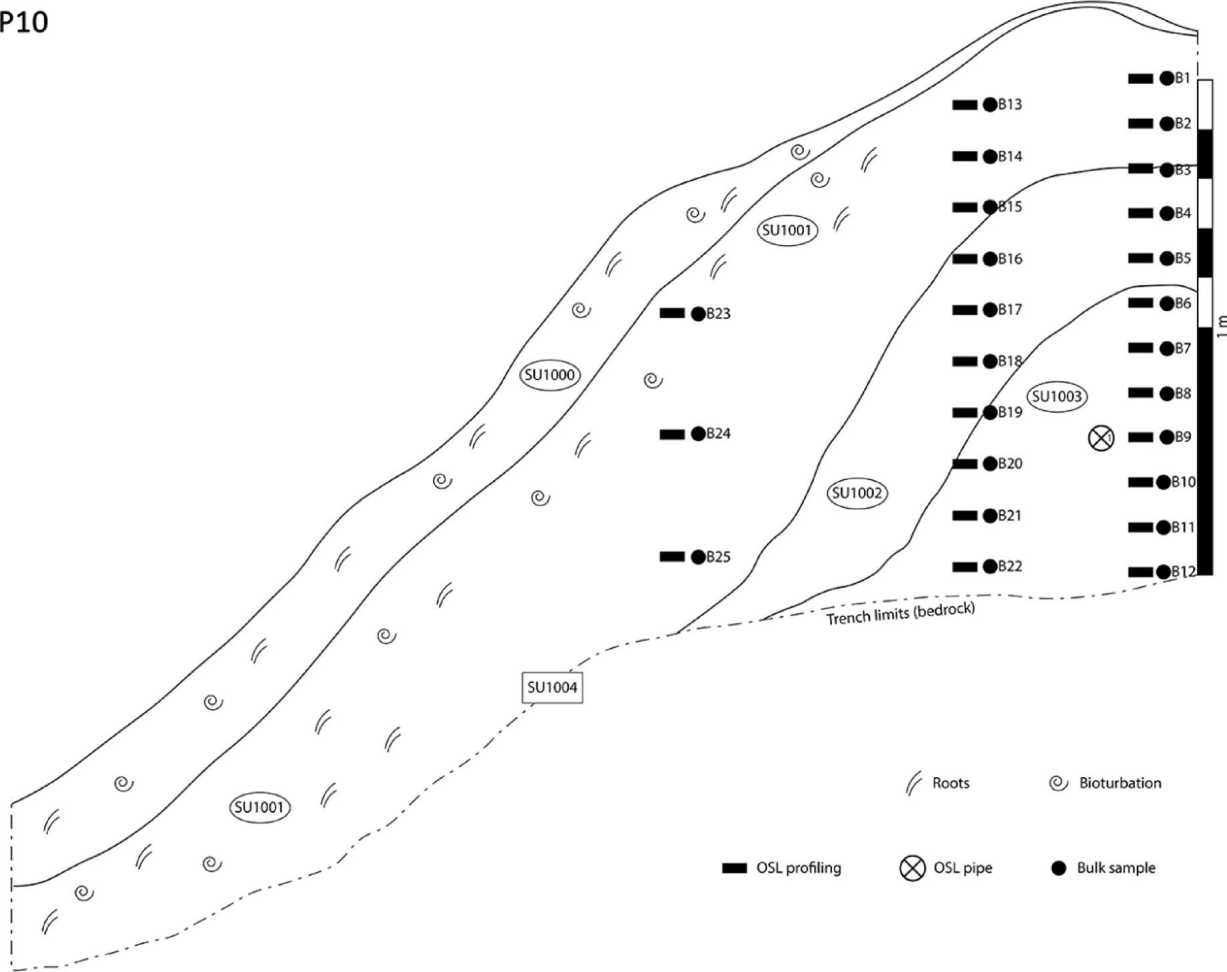
Stratigraphic section of profile number 10 (P10, *Pineto*). This linear feature is an earthen bank running downhill perpendicular to the slope’s contour lines, and the excavation drawing portrays one of two symmetrical halves. SU 1001: active organic litter, rich in gastropod shells and decaying plant matter; SU 1001: loamy deposit, cleared of stones and pebbles. Modest humification and bioturbation, occasional presence of gastropod shells. SU 1002: brittle loamy deposit, cleared of stones and pebbles; SU 1003: hard loamy deposit, cleared of stones and pebbles, constitutes the core of the earthen structure; SU 1004: lower limit of the earthen structure, coinciding with the solid bedrock (SC). The OSL sample indicated in this figure corresponds to CERSA889 (Table 1).

The terraced slope at Pineto is located 6.5 km east of Ferro and Castello (Figs. 1 and 3). The first profiles examined the sediments forming the lynchet that delineates the northwestern boundary to the terraced fields. A profile was taken through the centre of the lynchet (P10, Fig. 6): the top 17 cm forms the plough soil, then from 17 to 60 cm depth the sediment is characterised by a heterogeneous distribution in intensities, ranging between 1.97 and 4.38 × 10^6^ counts; between 66 and 73 cm, intensities drop off from 7.89 to 2.13 × 10^5^ counts, before returning to values in the range 1.73 × 10^6^ counts (Fig. 4). The lynchet overlies a substrate characterised by intensities 7.30 to 7.87 × 10^6^ counts. It appears that the lynchet overlies a buried soil, ~ 1.7 × 10^6^ counts, and that the core of the lynchet was disturbed at construction, 2.13 × 10^5^ counts, before being immediately covered by sediment cut from the adjacent landscape, > 1.97 × 10^6^ counts. The buried soil was dated to 420 ± 270 CE (CERSA889; Table 1).

A ~ 1.5 m tall terrace wall immediately downslope of P10 was sectioned for OSL-PD (P11). The sediment retained behind the wall showed a signal progression with depth from ~ 2.0 × 10^5^ counts beneath the ploughed soil to 2.82 × 10^5^ counts at 150 cm depth. Beneath this is the substrate, 6.07 to 6.79 × 10^6^ counts. A lower course of the wall survives at a depth equivalent to 150 cm depth in the stratigraphy: the sample taken from here dates to 1280 ± 110 CE (CERSA890; Table 1).

**Fig. 7:**
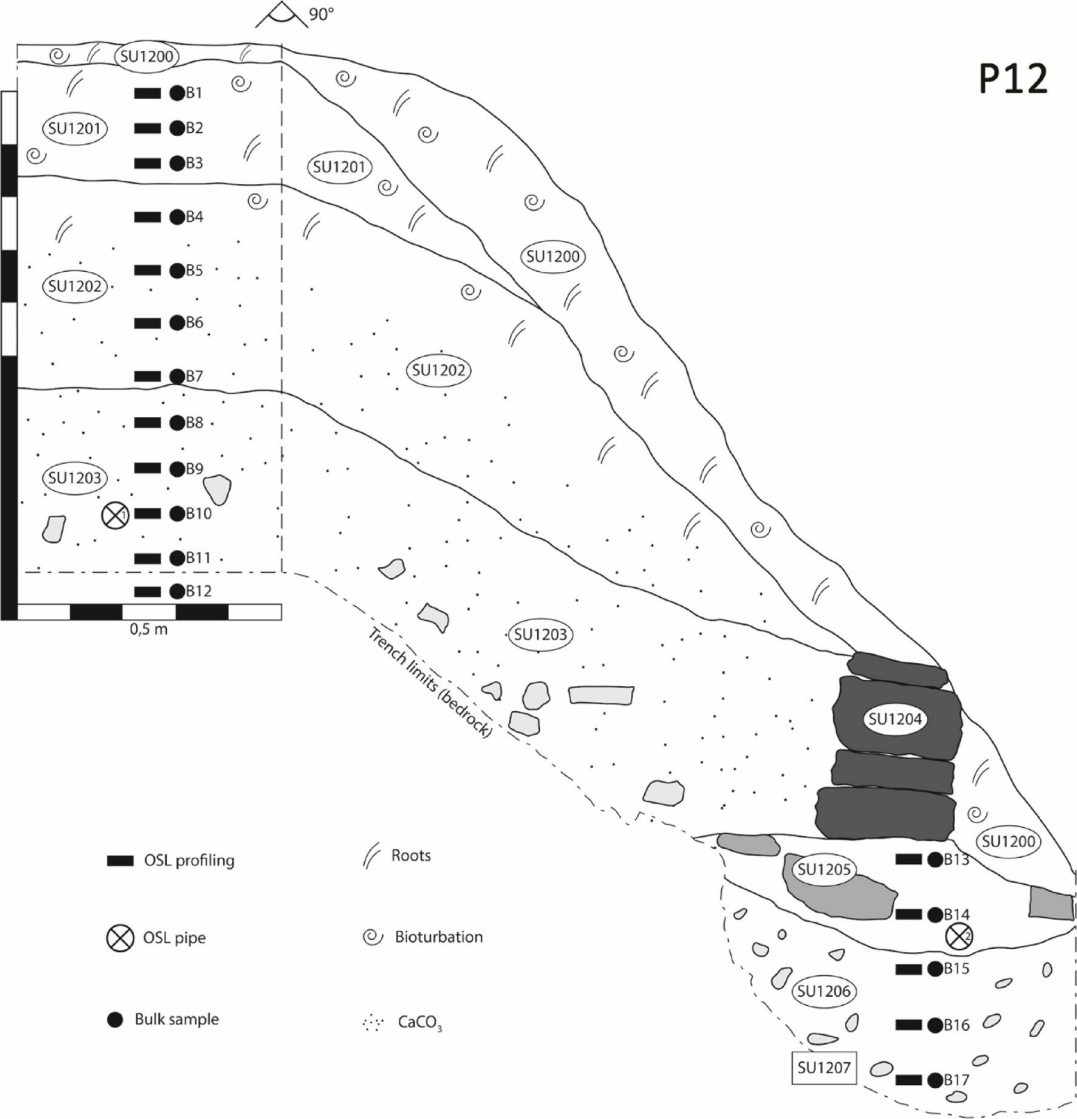
Stratigraphic section of profile number 12 (P12, Pineto). SU 1200: active organic litter, rich in gastropod shells and decaying plant matter; SU 1201: loamy deposit, cleared of large stones and pebbles, constitutes the cultivable terrace fill. Modest humification and bioturbation, occasional presence of gastropod shells. Level surface extends for a few metres behind the excavated trench; SU 1202: loamy deposit, cleared of large stones, with partial carbonate nodules impregnation in the lower half. Likely part of the cultivable depth; SU 1203: loamy deposit with small stones included in the lower half, with abundant nucleation of gravel-sized calcium carbonate nodules SU 1204: large oblate stones constituting the wall; SU 1205: compact loamy deposit containing occasional medium-sized and large irregular stones; SU 1206: hard loam with small irregular stones, likely naturally occurring regolith or anthropogenic deposit with very limited transport and alteration. SU 1207: lower limit of the terrace structure, coinciding with the solid bedrock. The bedrock’s surface underneath the wall was likely shaped as a foundation trench to host a levelling fill (SU 1205 and 1206) to ease the wall’s construction.

A further six trenches were positioned on the slope beneath P10, each investigating a further terrace wall. The sediments associated with the terrace walls were explored through 6 profiles. Two of these (P12 and P16, Fig. 7), revealed sediment chronologies similar to P10 and P11, with the base of the profiles characterised by intensities in the range 1.91 to 1.97 × 10^6^ counts - likely deposited in the 4th and 5th centuries CE, with the overlying soils, < 6.87 and 8.16 × 10^5^ counts, - likely accumulating in the 12th and 13th centuries CE. The remaining four were characterised by more complex distributions, with little stratigraphic dependence, with intensities varying between 1.28 and 9.92 × 10^6^ counts. The most likely explanation is that this sediment was not exposed to adequate light during transportation and deposition to reset the luminescence signals.

### Total organic carbon (TOC) content and δ^13^C

The analysis of the bulk TOC content and its isotopic fractionation yielded remarkably consistent results across both measurements (Table 3). TOC is attested to a mean value of 4.618% on weight, with a standard deviation of 0.29 and no outliers. δ^13^C has a mean value of −25.57‰, with a standard deviation of 0.61 and no outliers. This analysis was conducted in the Ferro area (P2, P3, P4 and P5) to investigate a potential correlation between the temporal control provided by OSL dating and Land Use and Land Cover (LULC) changes. The results confirm that the dominant control on signal intensities is age (the soil geochemistry and bulk sediment characteristics also support this).

**Table 3:** δ^13^C values for the bulk samples collected at Ferro.

	Trench	Bulk sample	TOC (w%)	δ^13^C	TOC Mean/ Standard deviation		δ^13^C Mean/ Standard deviation	
Ferro	P2	1	4.500	−26.182	4.434% 0.254	4.618% 0.29	−25.42‰ 0.52	−25.57‰ 0.61
		2	4.408	−25.82				
		3	4.402	−25.204				
		4	4.302	−25.414				
		5	4.295	−25.612				
		6	4.169	−25.114				
		7	4.957	−24.567				
	P3	1	5.565	−26.825	4.840% 0.340		−25.97‰ 0.41	
		2	5.082	−26.33				
		3	4.650	−25.856				
		4	4.715	−25.954				
		5	4.611	−25.63				
		6	4.610	−25.776				
		7	4.586	−25.659				
		8	4.899	−25.739				
	P4	1	4.722	−26.263	4.527% 0.128		−25.98‰ 0.21	
		2	4.435	−26.022				
		3	4.399	−26.048				
		4	4.542	−25.799				
		5	4.538	−25.748				
	P5	1	4.703	−24.635	4.610% 0.175		−24.74‰ 0.20	
		2	4.302	−24.519				
		3	4.698	−24.729				
		4	4.634	−25.068				
		5	4.712	−24.726				

## Discussion

This study has produced valuable information about the Late Holocene agricultural, societal, and landscape transformations that occurred in the study area.

**Fig. 8:**
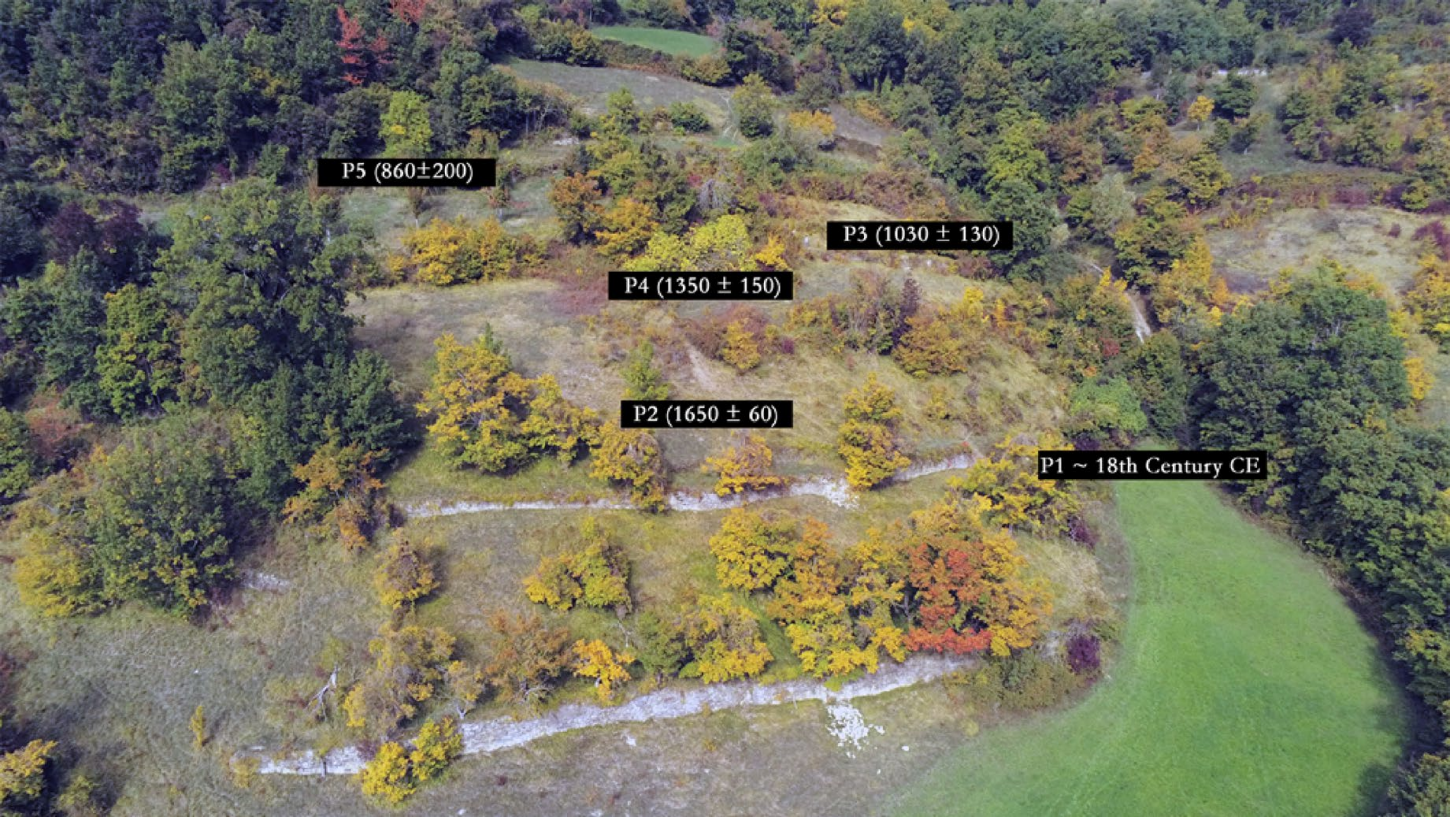
OSL constraints for construction of the terraces in the Ferro area. The dates of the earliest construction phases of the historic terraces reveal a distinct correlation between the age of terrace construction and altitude.

Firstly, it is noteworthy that the chronologies of the historic terraces in the area display a clear relationship between age and altitude, with the earliest constructional phases observed at the highest elevations at Ferro, Castello and Pineto (P5, P7, P10), particularly evident at Ferro (Fig. 8). The geomorphological dynamics of terrace farming were not a primary focus of this research, but this age-altitude relationship warrants mention and brief discussion. This pattern likely results from the increased mechanical stress on the lower terraces, which bear the cumulative load and impacts of slope geomorphic processes affecting the terraces higher up the hillside.

**Fig. 9:**
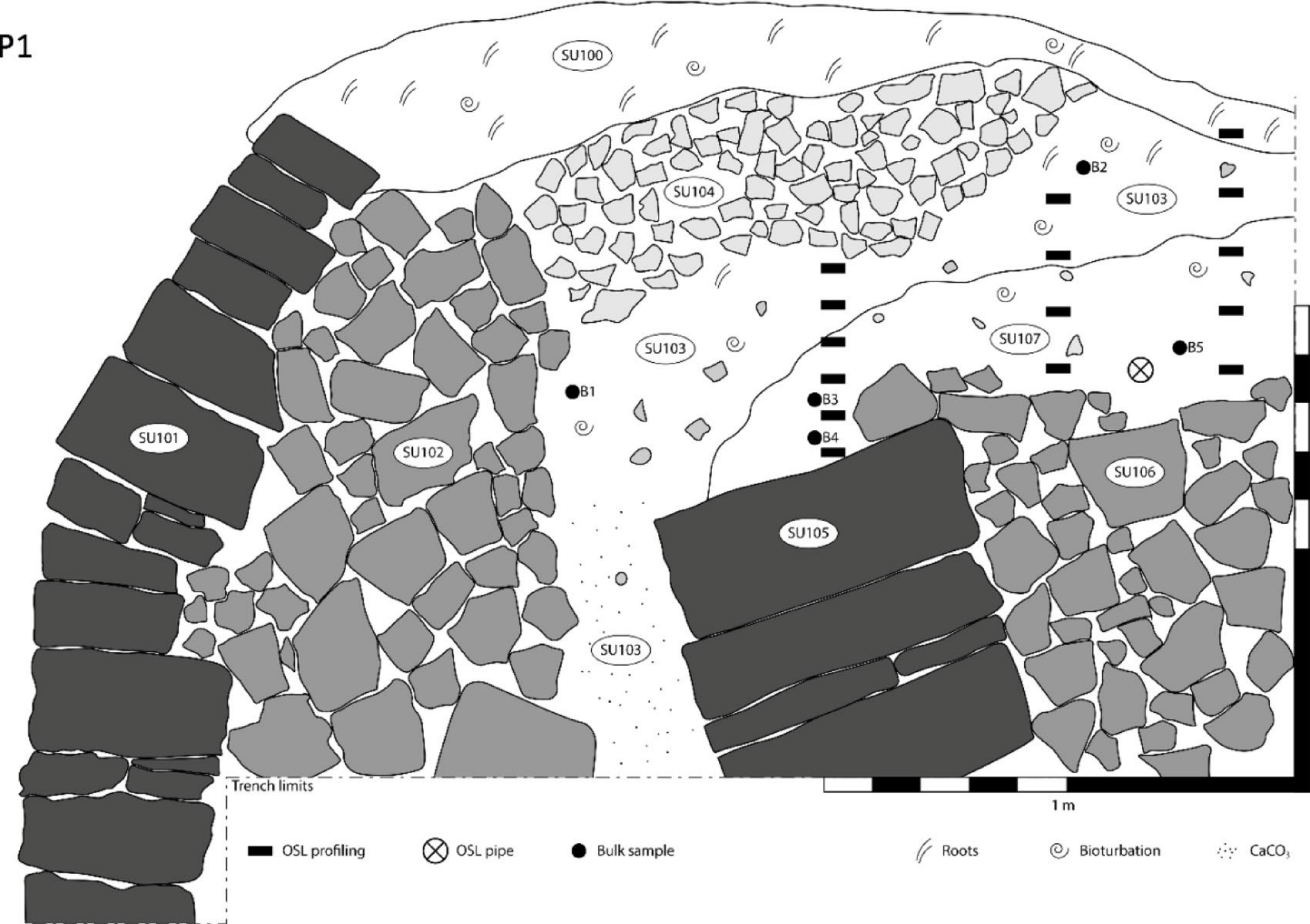
Stratigraphic section of Profile 1 (P1, *Ferro*). SU 100: active organic litter, rich in gastropod shells and decaying plant matter; SU 101–104: constituents of the outer terrace structure. Small and medium-sized stones, together with cleared loam, are used to create mass for the enlargement of the walkable surface. Large cuboid stones are tilt-piled to provide enhanced dissipation of the weight and mechanical stress. SU 105–107: constituents of the collapsed inner terrace structure. The stones used for the walls were very large, but a probable lack of counter tilt failed to provide resistance to the mechanical stress induced by naturally occurring mass waste and soil creep.

Terraces that have been abandoned or are not maintained quickly accumulate sediment, with their walls tending to collapse due to factors such as earth pressure and soil creep^39^. Partial collapse of terraces is often attributed to increased earth and water pressures behind the inner face of the retaining wall, resulting from gradual mass wasting and inadequate water drainage along the terraces^15^. Consequently, our hypothesis is that, over time, more frequent restoration efforts may be required on the stone walls of the lower terraces, which are subjected to the weight of the entire hillside section. This phenomenon might explain the distinct phases observed in the construction of wall P1, where a newer wall was constructed atop an older one that toppled facing downhill (Fig. 9), a situation not observed in the terraces higher up in the same sequence (Fig. 8).

**Fig. 10:**
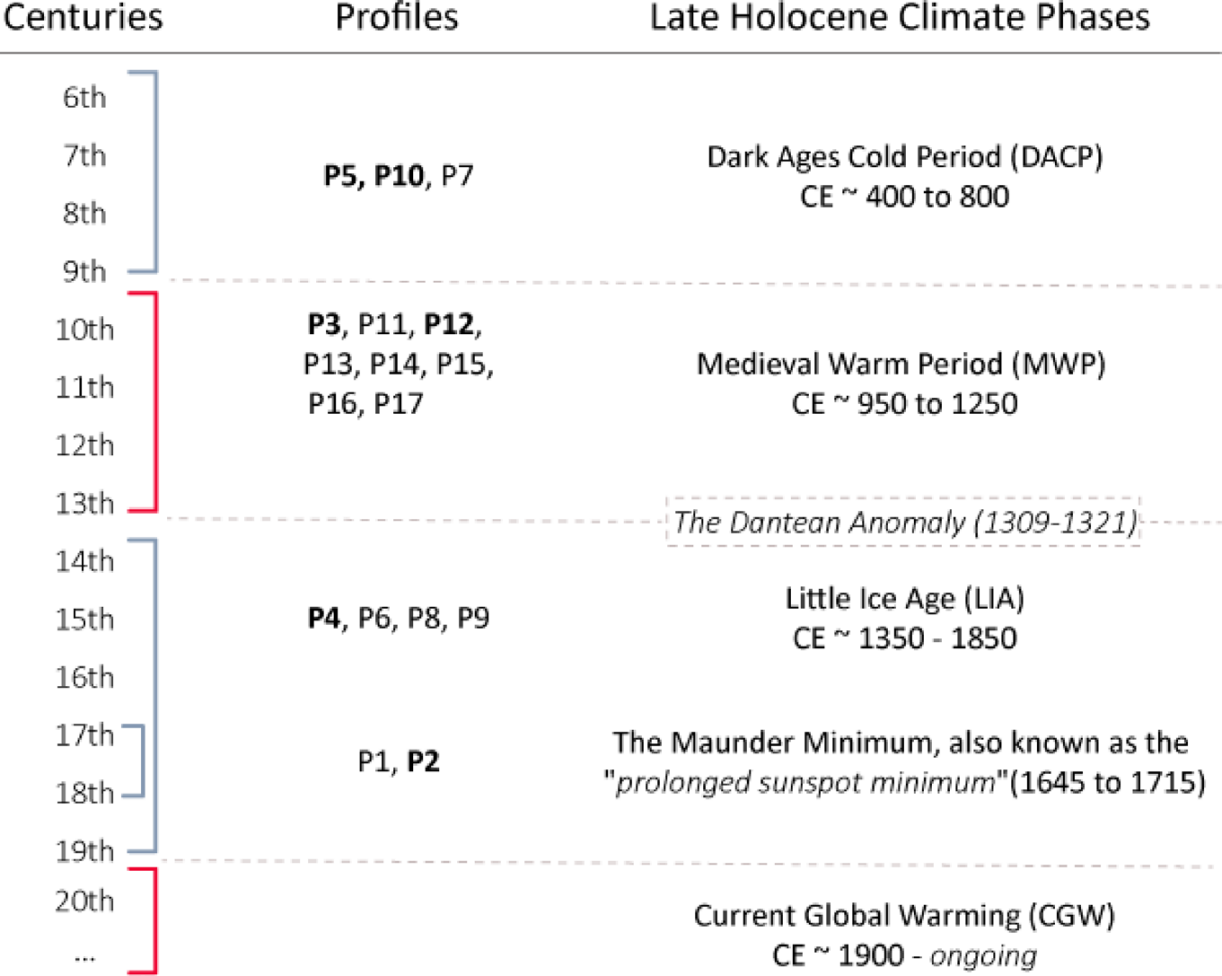
A temporal framework for the profiles sampled in Vetto (profiles with OSL absolute chronologies in bold) compared with the primary Late Holocene climatic variations. Warm periods are indicated in red, and cold periods are indicated in light blue.

The temporal framework defined for Vetto, with the earliest phases dated to the 9th century CE, followed by an intensification of terrace and earthwork construction between the 11th and 14th centuries CE, establishes this area as one of the oldest known examples of terrace farming systems in northern Italy. The intensification phase (11th – 14th century CE) falls within the Late Holocene climatic epoch, referred to as the Medieval Climate Anomaly (MCA), and the transitional period leading to the subsequent climatic fluctuations of the Little Ice Age (LIA). In the northern Hemisphere, the MCA signifies the most recent natural warm phase, spanning roughly from around 950 to 1250 CE. It is preceded by the Dark Ages Cold Period (DACP, ca. 400–800 CE) and succeeded by the LIA (ca. 1350–1850 CE)^40^ (Fig. 10). This correlation between the intensification of terrace farming and the MCA raises insights into human adaptations to past climate changes within the study area. MCA warming predominantly influenced the Western Mediterranean and the northern terrestrial zones of the Central and Eastern Mediterranean region, with a core span from 1000 to 1200 CE, and it is characterised by above-average temperatures that may have facilitated the continuity of high-elevation settlements^41^. The TOC content and isotopic fractionation of the soil in the study area suggest processes influenced by climate variations, supporting the temporal framework established for Vetto.

The TOC measured in the bulk samples, averaging 4.6% on weight at all depths, indicates translocation and fixation of soil organic compounds driven by modest pedogenesis in a temperate moist climate^42^. The δ^13^C mean value of −25.57%, moreover, falls perfectly in the value bracket associated with temperate C3 vegetation (δ^13^C: −20‰ to −35‰)^43^, providing a general hypothetical picture of an agronomic land-use characterised by broadleaf open canopy associated with temperate cultivars such as wheat and barley. Interestingly, however, bulk samples from P5 show a mean value of −24.74‰, which fall outside of the total standard deviation calculated from the values of the entire terrace system at Ferro. While this value is still largely within typical C3 vegetation brackets, it is worth noticing that this terrace is the oldest from the sequence, with an age that is only slightly younger than the termination of the DACP. Although our available soil composition data is currently limited, one possible explanation for P5’s lower values is that they may have been influenced by an extended local presence of European-native C4 plants (δ^13^C: −9‰ to −16‰)^44^ adapted to temperate climate with intermittent dry spells. A viable example of such plants is the Boraginaceae family^45,^ a C4 plants family that comprises some synanthropic species that are edible for human consumption (e.g. *Borago officinalis*), which are fast-spreading over rocky terrain and well adapted to open environments and cold dry spells. Unfavourable conditions during the DACP might have resulted in reduced maintenance of the upper parts of the *Ferro* terrace system leading to a fast colonization of the area by such plants and other weeds, ultimately registered in lower soil δ^13^C values.

Further considerations relate to historical socio-cultural dynamics (see ‘Material and Methods’). In the study area, the precise origins of Canossa’s castles remain uncertain, with their dates for construction reliant on information obtained from medieval chronicles. Historical records indicate that most of the castles were established between the 10th and 11th centuries CE^46^ although archaeological evidence only sporadically confirms this chronology^47,48^ with the earliest discernible phases date back to the 12th and 13th centuries CE^49^. Vetto itself was first recorded in 1142 CE and historical evidence suggests a castle was built here in 1185 CE, though these dates serve only as the *terminus ante quem* for the establishment of the medieval settlement. Thus, the findings from the OSL-PD analysis provide new insights into the medieval history of this segment of the northern Apennines and the consequences of socio-cultural dynamics on the landscape. Firstly, the presence of agricultural terraces in the region suggests the possible existence of stable Early Medieval exploitation dating back to at least the 9th century CE during the latest phases of the DACP (as indicated by P5 and P7). This raises questions about the role that local peasant communities played in shaping the agrarian landscape of the area. Studies of Early Medieval agrarian systems have emphasised that agricultural production was often decentralised, with local communities actively involved in organising and intensifying farming practices^50^. As reported in other European regions, terrace farming likely emerged not only from external pressures, such as climate change or feudal power dynamics, but through the proactive engagement of peasant communities who were essential actors in forming their agrarian landscapes^51^​. It seems most likely that terraces and other agricultural spaces are not merely products of top-down demands but represent intentional, community-based strategies for managing local environmental and economic conditions^52,53^. Rather than being solely a response to political directives, it is plausible that these terraces reflect peasant-driven agricultural intensification, which may have contributed to establishing Vetto as a productive and strategically valuable site even before the advent of the Canossa rulers. Similar processes of landscape transformation have been documented in other parts of Early Medieval Europe, where agrarian space organisation often resulted from community-driven initiatives rather than top-down imposition^54^. Geoarchaeological studies in northern Iberia, for example, have highlighted how peasant communities played an active role in reshaping rural landscapes through coordinated efforts, including terrace construction and soil management, as part of broader social and economic transformations^55^.

Secondly, it is well-known that the fortified system of Canossa played a pivotal role in safeguarding the principal routes that connected the Po-Venetian plain to central Italy across the Apennines^56^ but the impact of the encastellation process on the rural landscape in the area is still poorly explored. As observed on the Ligurian^11^ and central Apennine mountains^57^ it is plausible that during the MCA even in the Canossa Kingdom, the establishment and renovation of settlements in more defensible elevated locations was accompanied with deforestation, and intensification of agricultural activities. The chronologies of terraces from the profiles sampled at Castello and Ferro support this interpretation since a sophisticated terrace farming system was established around the village during the 11th century CE and even before the construction of the castle which occurred in the late 12th century CE. This chronological sequence of events implies that the construction of the terraces was unlikely to be a result of the prominent role achieved by Vetto through the establishment of the stronghold. Alternatively, the evidence might suggest that the site had already held a significant status within the Canossa Kingdom framework. The creation of agricultural terraces, which required substantial economic and human resources, could indicate that the Canossa house’s activities in the area extended beyond fortification, involving a broader reorganisation of the rural landscape and agricultural strategies. As known from historical records, the population in northern and central Italy grew substantially during the MCA, increasing the demand for cultivated lands and leading populations to reconfigure the medieval natural landscape for agricultural purposes^58^. This general phenomenon that also affected other European regions might explain the adoption of agricultural terraces on the northern Apennines during the MCA. Additionally, the chronologies derived from the samples indicate that the area under terrace farming continued to expand even during the 13th century CE. It is likely that the landscape reorganisation by the Canossa rulers triggered an intensification in the use of terrace farming in the region. Meanwhile polygonal masonry required substantial communal labour and the more centralised control of the territory during the Canossa Kingdom likely facilitated the spread of this technique in the area. Therefore, the emergence of terrace farming in this specific section of the northern Apennines seems to be the outcome of a multifaceted interplay between socio-cultural factors (encastellation process) and economic necessities (demand for additional cultivable land), linked to changing climatic conditions during the MCA. As in other geographical and archaeological contexts^4^ even in the study area, climatic changes likely stimulated technological and societal innovations.

The chronological framework constructed for the Ferro and Pineto slopes helps to shed light on the later evolution of this landscape. The dates from these profiles likely correspond to phases of revitalizing the medieval terraces in the area. Although it was not possible to reach the bottom of the sequences, the OSL-PD results for P6, P8, and P9 suggests that the earliest these could date to is the mid-14th century, possibly representing the oldest restoration of these terraces rather than their exact moment of construction. Moreover, the lynchet from which the P4 samples were taken could potentially be identified as a ‘*ciglione*’, a characteristic earth feature used extensively in the rural landscape organisation of central and northern Italy during the Renaissance^59^. ‘*Ciglioni*’ served multiple functions in the agricultural landscape, preventing soil erosion on hillsides, creating level surfaces suitable for crop cultivation, and facilitating improved water management through the construction of lynchets with varying elevations, effectively capturing and distributing water^60^. Dendrochronological records from the 14th century CE across Europe indicate wetter summers with an initial cold spell around ~ 1300 CE as the earliest manifestations of the LIA^58^ known as the Dantean Anomaly or the “1310s event”^62^. Recent studies have demonstrated that this sudden wet and cold transition period between the MCA and LIA (1309–1321 CE) presented immediate ecological stresses to societies throughout Europe^63^. An increase in precipitation could have prompted the restoration (P6, P8, P9) and improvement (P4) of the terrace farming system in the study area. This enhancement might have involved the construction of lynchets (P4), aimed at reducing downslope soil erosion caused by runoff, a persistent environmental concern that continues to impact the study area^14,17^.

P1 and P2 correspond to the most recent phases of the Vetto farming system that were sampled. Construction of P2 likely aligns with the Maunder Minimum (1645 to 1715 CE), the coldest phase of the LIA^64^. The cooler temperatures and shorter growing seasons had a significant impact on agriculture throughout Europe, with crop failures, reduced yields, and famine common in many regions^40,62^. During this timeframe, significant variations in solar irradiance occurred, leading to colder temperatures across the northern Hemisphere continents, particularly in winter, where temperatures dropped by approximately 1 to 2 °C^65^. The building of terraces at Vetto during the LIA can likely be attributed to two main factors: (1) the need to restore the stone walls, which might have been at risk due to the increasingly harsh environmental conditions; (2) the need to use sun-exposed slopes for agricultural purposes in order to mitigate the risk of crop failures. Finally, it is noteworthy that P1 was modified in the late 18th century CE^17^ which was traditionally considered to be when terraces were first established in the area^35^. By contrast, the archaeological evidence shows that it marks the last historic renovation phase of the Vetto terraces.

Similar chronological patterns have been recorded in other western Mediterranean case studies, with the most intense episodes of terrace-building during the MCA and subsequent renovation during the LIA^25^. Recognising this general trend is relevant to understanding the connections between ecosystems, social organisation and human adaptation to climate change during the Late Holocene. As a period characterised by natural, pre-industrial climate fluctuations which were marked by substantial temperature and hydroclimatic variation, research on the MCA provides interesting parallels for the ongoing Current Global Warm phase (CGW) and its impacts^64,66^. The episodes of construction and restoration indicated by the OSL-PD results, not only in this study area but also in other Mediterranean case studies^25^ emphasize the significance of these systems during periods of climate change including not only the MCA but also the subsequent cold phase of the LIA. This underscores the adaptability of terrace farming as a robust agricultural strategy capable of effectively responding to varying climates. Throughout history, societies have observed cycles of growth and decline, closely linked to environmental changes through impacts on aspects such as water availability and agricultural productivity^67^. Today’s societies are grappling with enormous challenges posed by the CGW to ecosystems and human health^68^. While modern economic systems may be more resilient to short-term climatic shifts than pre-industrial societies, they are not immune from long-term extreme alterations in temperature and precipitation patterns^61^. Indeed, projections already suggest northern Italy is vulnerable to the abandonment of floodplain farmland in the coming decades^69,70^. In this context, the implications of our research extend beyond the archaeological evolution of a rural landscape. The OSL-PD analysis revealed that terrace farming not only had significance as a historical practice but also served as a highly resilient and adaptable solution. Its revival within present economic systems might provide a sustainable agricultural strategy in response to the challenges posed by the CGW.

## Materials and methods

### Site selection

For site selection and identification of potential locations for geochronological sampling, field surveys were first carried out using remote sensing imagery and mapping^13^. The historic terraces were sampled at three distinct locations within the Vetto municipality: (i) Ferro, (ii) Castello, and (iii) Pineto (Fig. 1). These three localities were selected based on factors related to representativeness, historical and modern disturbance, ease of access, and impact on present land use. Trenches were excavated to reveal sediment stratigraphies which were protected under temporary opaque tarpaulin cover. A total of 17 profiles (both terrace walls and earth banks) were investigated in these three study areas: Ferro (3 terraced walls, 2 earth banks/lynchets), Castello (4 terraced walls) and Pineto (7 terraced walls, 1 earth bank/lynchet) (Fig. 3).

### Historical background

In the late 19th century CE a limited number of ceramic fragments tentatively attributed to the Bronze Age were collected in the study area^71^. While the name ‘Vetto’ is commonly believed to have Roman origins (from latin ‘*Vectus*’, likely referring to the area’s strategic crossing over the Enza River)^72^ its first historical mention dates back to the 12th century CE. In a document dated 1142, Vetto is specifically referenced in border disputes between the dioceses of Parma and Reggio Emilia, along one of the main routes connecting the Po Plain with Tuscany^73^. Between the 10th – 12th centuries CE, Vetto underwent a process known as ‘*incastellamento*’ (encastellation). This process was widespread in Italy and has traditionally been attributed to attempted mitigation of threats posed by Muslim, Magyar and Norse invaders through the construction of fortified castles (which often comprised a motte with earthen ramparts, wooden fences and a ditched perimeter)^74,75^. The creation of fortified positions throughout the northern Apennines formed part of a broader phenomenon observed in many parts of Europe from the 10th century CE onwards. Initially, the regent of the Italic Kingdom held exclusive authority to grant local lords the privilege of erecting fortified structures. However, following the decline of royal control in Italy around the mid-10th century CE, numerous influential noble families began constructing their own castles independently^76^. During the 11th century CE, political authority in northern Italy became dispersed among numerous local entities, with profound social and cultural implications. In the Emilia-Romagna and Tuscany regions, the Canossa rulers led the reorganisation of the landscape with the construction of both defensive and ecclesiastical buildings^46^. The ascendancy of the Canossa family’s political influence commenced with Adalberto Atto of Canossa (977–984 CE) and culminated during the rule of his great-granddaughter Matilda of Tuscany (1052–1115 CE), renowned as the Great Countess and hailed as one of the most impactful figures of her era^77^. The Great Countess Matilda enriched the defensive network of Canossa not only by renovating existing castles but also by likely erecting new ones^56^. Starting from the 12th century CE, Vetto fell under the control of the earls Da Palude, a prominent family allied with the counts of Canossa. In 1185, the Da Palude family obtained permission from the Holy Roman Emperor Frederick I to build a stronghold in Vetto. This castle was subsequently dismantled by the city of Reggio Emilia in 1315 but was partially reconstructed in the area known as Castello, which corresponds with the oldest nucleus of Vetto^73^. There is limited information available for the following centuries. Between the 14th and late 18th centuries CE, the area of Vetto was under the control of the House of Este^73^ and the area’s historic landscape appears to have remained largely unaltered from the mid-19th century CE until the end of the 1950s^13^.

### Geology and stratigraphy of the terraces

The municipality of Vetto d’Enza gives name to the local dominant lithostratigraphic unit Arenarie di Vetto (following sheet 218 of the Carta Geologica d’Italia^78^ 2002). The unit is composed of Miocene bedded calcilithic arenite with pelite layers, light yellowish-grey in colour, with a same-aged glauconite level and debris slumps close to the top. Unaltered hillslopes are blanketed with in-situ weathered bedrock and quiescent landslides, both producing a loamy regolith characterised by a matrix-supported deposit with chaotic inclusion of heterometric clasts. In correspondence with the terraced slopes, the components of the regolith were selected and separated for terrace engineering: large, squared stones were used for the outer walls, while progressively smaller and irregular stones were accumulated behind the outer walls for reinforcement; fine matrix, cleared of stones, was used to fill the gaps and level the step to create walking paths and cultivable surface (Fig. 5).

The stratigraphy of the terraces and banks across the sites (Ferro, Castello and Pineto) is remarkably consistent, with almost no intra-site and inter-site variability. Slight differences are only dictated by bumps and slope variations in the bedrock, which in turn condition the depth and width of each terrace, the choices regarding the size and number of stones used for wall construction, and the presence of woodland-induced taphonomic disturbances (e.g.: pedoturbation caused by large roots, composition variability and thickness of the surface organic litter) (Fig. 5).

### Geochronology

A preliminary chronology was reported by Brandolini et al. 2023^17^ within the framework of a diachronic soil erosion modelling study, evidencing the ancient origins of these landscape features. The uniqueness of this research lies in its novel approach towards understanding the genesis and evolution of terrace farming in the area, expanding our insights from a single site to an intra-site scale. This has been accomplished by integrating information from each trench through geochronological and pedosedimentary analyses and aligning the resulting periodisation with the historical and socio-cultural context of the area.

The depositional ages obtained for the sediments associated with the Ferro terraces are reproduced in Table 1. To expand the chronology for terrace construction at Ferro to the wider historic landscape of Castello and Pineto, we contextualise the luminescence stratigraphies constructed at Ferro, Castello and Pineto within the framework of luminescence ages reported in Brandolini et al. 2023^17^. These additional stratigraphies were generated using the methodology of OSL-PD, as suggested by Kinnaird et al. 2017^24^ and Turner et al. 2021^25^. In this approach, the luminescence behaviour of bulk sediment is appraised using portable OSL equipment, coupled with in situ gamma spectrometry. Immediately after excavation, the trenches were covered by opaque black tarpaulins, with final cleaning before sampling for OSL undertaken beneath these. Bulk sediment samples were taken at regular intervals down-profile and screened on site using portable OSL equipment^37^ using an interleaved sequence of IRSL, OSL and background. This information was used to calculate IRSL and OSL net signal intensities, IRSL and OSL depletion ratios and IRSL: OSL ratios, which were plotted against depth, and reviewed in the field, so that the stratified sequences were considered with a time-depth component. In-situ field gamma spectrometry measurements were taken throughout the sediment sequences using a Gamma Surveyor Vario coupled with a 19 cm^3^ Bismuth Germanate Oxide detector.

To validate the relative chronological framework established for the sediments at Ferro, a subset of the samples collected and screened in the field were progressed to laboratory analysis – stage 2 in the methodology of Turner et al. 2021^25^. This served two purposes: one, to check the temporal and spatial inferences drawn from the field profiles; two, to expand the investigations into the adjacent strata.

For Stage 2 laboratory analysis, from each sample (collected for OSL-PD in the field), a sub-sample of 90–250 microns was isolated using wet-sieving and was treated with hydrochloric acid (HCl) and hydrofluoric acid (HF) to obtain ‘quartz’. All of the laboratory preparations were conducted in subdued orange light conditions at the luminescence laboratories in the School of Earth and Environmental Sciences at the University of St Andrews, and all OSL measurements were carried out using a Risø TL/OSL DA-20 automated dating system, equipped with a ^90^Sr/^90^Y β-source for irradiation, blue LEDs emitting around 470 nm and infrared diodes emitting around 830 nm for optical stimulation^79^. OSL was detected through 7.5 mm of Hoya U-340 filter and detected with a 9635QA photomultiplier tube. A set of two aliquots of these ‘quartz’ were mounted on a stainless-steel disc (as a small aliquot) and were subjected to a standard SAR protocol^76^ and determined stored doses were plotted against depth and compared with luminescence sensitivities generated in field.

In relative time-depth, the construction of terrace wall P1 would have occurred in the 17th century CE i.e. the sediment beneath the wall returns intensities < 3.1 × 10^5^ counts (see discussion in main text). The apparent dose-depth profile for P1 follows a similar trend to that observed in the OSL signal intensity-depth plot, albeit with some heterogeneity – justifying the approach outlined here. To validate this, one of the samples selected for stage 2 screening, CERSA858-3 at 59 cm depth in profile 1, was progressed to further laboratory testing: 24 small-aliquots were dispensed to disc and subjected to a full quartz SAR OSL procedure. Many of the aliquots failed the rejection criteria in the SAR protocol, but those that passed produced a burial dose of ~ 1 Gy, which together with the environmental dose rate of 2.5 ± 0.1 Gy ka^-1^, corresponds with a depositional age in the ~ 17th century CE, as originally proposed. In relative time-depth the earthwork at P2 would also have been constructed in the 17th −18th centuries CE i.e. the sediment at the base of the bank returns an intensity of 3.1 × 10^5^ counts, whereas at the interface between bank and substrate, 7.8 × 10^5^ counts. Again, the apparent dose profile follows a similar trend to that observed in OSL signal intensities: the substrate beneath the bank returns apparent doses > 5.9 Gy, whereas the bank returns apparent doses in the range ~ 1.1 to ~ 0.5 Gy. The environmental dose rates estimated at 0.8 and 0.9 m depth in profile are 2.5 and 2.4 Gy ka^-1^, would correspond with depositional ages from the mid-17th centuries CE and later. This provides further corroboration for the relative temporal framework suggested in the main text. The interface between the bank and substrate is readily apparent – showing that it is possible to distinguish the different sediment packages based on the stage 1 data.

**Fig. 11:**
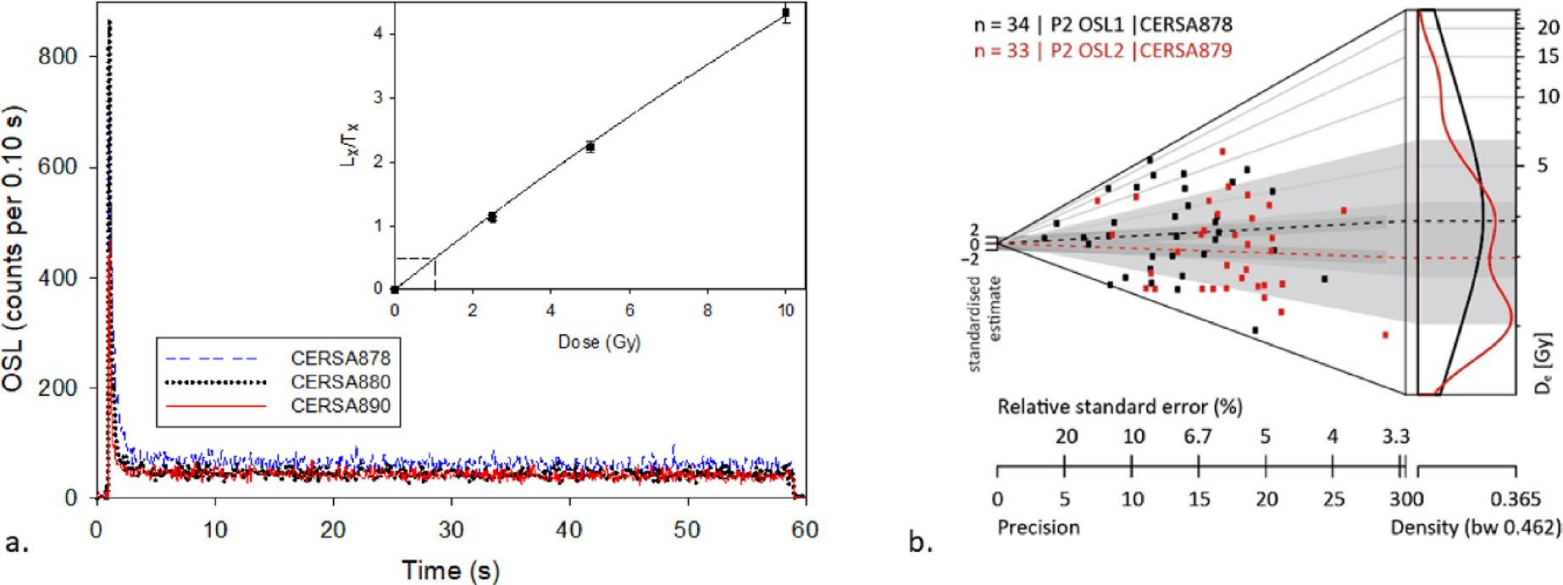
(**a**) Representative decay curves for small aliquots of samples CERSA878, CERSA880 and CERSA890 together with a corresponding dose response curve for CERSA878 (inset) fitted using an exponential function. (**b**) Abanico plot showing dispersion in individual D_e_s for the samples CERSA878 and CERSA879. (modified from Brandolini et al. 2023^17^).

For Stage 3 which involves determination of absolute OSL ages, more rigorous laboratory procedures were applied. Sediments from the middle of the tube were wet-sieved to extract 90–250 μm sized particles followed by treatments with HCl and hydrogen peroxide (H_2_O_2_) to remove carbonate and organic matter respectively^24,80^. Samples did not show any visible reaction with HCl suggesting low levels of carbonates but showed visibly mild reaction with H_2_O_2_. Quartz mineral grains were removed from the bulk sample using LST heavy liquid density separation which was followed by chemical etching using hydrofluoric acid to remove the alpha-irradiated outer surface of the quartz grains. and remove any persistent feldspar contamination. Samples from Pineto (CERSA 889 and 890) were additionally treated with fluorosilicic acid for two weeks^80^. After each chemical etching, HCl was used to remove any fluoride precipitate, and samples were sieved to isolate the 90–150 μm sized particles, fully prepared dry quartz was mounted onto stainless steel discs as a 2 mm diameter (small aliquot) monolayer using Silkospray silicone oil. D_e_s were determined using a SAR protocol with a pre-heat of 220 °C for 10 s and OSL measurement at 125 °C for 60 s, with the signals integrated over the first 1 s of stimulation, minus a late background over 10 s. The protocol was derived by performing dose recovery tests first. The regenerative dose response was constructed using a range of four to five regenerative doses to enclose the natural dose (Fig. 11a) with an additional zero dose, a repeat recycling dose and an IRSL recycling dose.

Data reduction and De determinations were made in Analyst v.4.31.9^81^ and the package Luminescence in R^82^. Dose response curves were fitted with an exponential plus linear function, with the growth curve fitted through zero and the repeat recycling points. Error analysis was determined by Monte Carlo simulation. Aliquots satisfying the following criteria were accepted for assimilation of Des: (1) recuperation of less than 5%; (2) recycling ratio within 10% of unity, including uncertainties^83^; (3) OSL IR depletion ratio within 10% of unity^84^ and (4) test dose signals 3σ greater than background levels. Luminescence behaviour of investigated samples was moderate to good, with small aliquots displaying bright signals (Fig. 11a). However, many aliquots measured failed to satisfy at least one of the rejection criteria outlined earlier and were excluded from dose distribution analysis. Up to 44 aliquots were measured per sample, and De determinations are based on at least 20 accepted aliquots.

Overdispersion or heterogeneity in the measured De distributions is relatively high in all the samples, ranging between ~ 40 to 100% (including uncertainties; Fig. 11b). These values, considering the geoarchaeological context of the sampled terraces, are not surprising and are along the lines of published values from other comparable studies^25,26^. Though heterogeneity in De distributions in terrace sediments can arise from a combination of different factors, two external factors i.e. partial bleaching prior to deposition and post-depositional mixing of sediments are considered the main reasons. The low overdispersion values observed in the dose recovery tests suggest that internal factors e.g. dosimetric variability and error in the instrumental sources, contribute little towards this heterogeneity. To derive a meaningful equivalent dose from such over dispersed data, the logged minimum dose model (MDM) of Galbraith et al. 1999^85^ was used. MDM assumes that only a proportion of measured doses belongs to the burial dose distribution and that the remaining dose estimates are part of a log-normal distribution truncated at the burial dose. MDM is considered appropriate here with the assumption that partial bleaching is the dominant cause of overdispersion as dating samples were collected for seemingly undisturbed strata.

Dose rates (Ds) were calculated from radionuclide concentrations (^232^Th, ^238^U and ^40^K) measured using Inductively Coupled Plasma Mass Spectrometry and Optical Emission Spectrometry at X-Ray Mineral Services Ltd, UK. These radionuclide concentrations were converted into infinite matrix Ds using the conversion factors of Guerin et al. 2011^86^, and then adjusted for attenuation by grain size and chemical etching using the datasets of Guérin et al. 2012^87^ and Bell 1979^88^ respectively. Ds were then corrected for sediment matrix water content as well (10–25% based on individual sample). Following Prescott and Hutton 1994^89^, the contribution from the cosmic dose (Dcosmic) was determined from the sections longitude, latitude and altitude, and the samples depth in section. All dose rates (Ds) calculations were made using the DRAC (v1.5) software of Durcan et al. 2015^90^.

### Isotopic fractionation of the total organic carbon (TOC)

The trenches selected for the analysis of the isotopic fractionation of the TOC were chosen from the Ferro sequence due to the consistency obtained from the OSL dating and the overall good preservation of the terraces’ walls and internal structures. Specifically, P2, P3, P4 and P5 were selected, yielding respectively 7, 8, 5 and 5 bulk samples of soil collected from the cleaned sections at evenly spaced ca. 10 cm intervals. Prior to chemical analysis, all visible modern organic debris (e.g. small roots) was picked with steel tweezers under a stereoscopic magnifier. ~ 1 g of each bulk sample was ground in an agate mortar rinsed with double-distilled water between each use. Ground bulks were then soaked in excess HCl 18% solution inside glass beakers for 48 h to remove the CaCO_3_ contribution from the subsequent measures. The suspensions were then transferred into 50 ml polystyrene Corning^®^ Falcon test tubes and rinsed with double-distilled water until neutral pH was reached, with the aid of a 6000-rpm centrifuge to speed up decanting between each rinse. Samples were then oven dried at 40 °C. Aliquots for the Thermo Fischer Flash 2000 Organic Elemental Analyzer coupled with a Thermo Scientific Delta V Advantage mass spectrometer via a ConFlo IV interface were prepared weighting ca. 10 mg and 4.5 mg of each sample within tin foil capsules using a nanogram scale in a locked isolated room. The 10 mg aliquots were destined to the measure of TOC values. The 4.5 mg aliquots were destined to the measure of the C isotope composition (δ^13^C). Carbon concentration was calibrated using aspartic acid (C = 36.09 ± 0.27 wt%); the international high organic sediment standard (δ^13^CTC = − 28.85 ± 0.10‰ 2σ; Elemental Microanalyses Ltd., Certificate No. 295716) and the international low organic soil standard (δ^13^CTC = −22.88 ± 0.40‰ 2σ; Elemental Microanalyses Ltd., Certificate No. 324704) were employed for the determination of the C isotope composition.

## Electronic supplementary material

Below is the link to the electronic supplementary material.

Supplementary Material 1

## Data Availability

The results of the geochronological analysis are available as supplementary materials attached to this paper.
