# Supplementary material for: Geoarchaeology reveals development of terrace farming in the Northern Apennines during the Medieval Climate Anomaly
Source: Sci Rep. 2025 Jul 10;15:24989. doi: 10.1038/s41598-025-08396-2 (PMC12246183; doi:10.1038/s41598-025-08396-2)
Supplement: Supplementary file 1 — Supplementary Material 1 [file 41598_2025_8396_MOESM1_ESM.docx]

Table S1: IRSL and OSL net signal intensities, IRSL and OSL depletion indices and the IRSL : OSL ratio for all bulk sediment samples. Colour shading in the ‘OSL signal intensities’ column reflects relative signal strength: cool colours indicate lower intensities (more recently bleached sediments), warm colours indicate higher intensities (older or poorly bleached sediments). This visual gradient helps identify anomalies and corresponds to the trends shown in the main figures.

| Field ID | Depth /cm | IRSL signal intensities / counts | IRSL depletion | OSL signal intensities / counts | OSL depletion | IRSL : OSL ratio |
| --- | --- | --- | --- | --- | --- | --- |
| P1-1 | 40 | 28010 ± 170 | 1.31 ± 0.02 | 191850 ± 440 | 1.48 ± 0.01 | 0.1460 ± 0.0009 |
| P1-2 | 47 | 48930 ± 230 | 1.35 ± 0.01 | 370230 ± 610 | 1.53 ± 0.01 | 0.1321 ± 0.0006 |
| P1-3 | 59 | 53360 ± 230 | 1.39 ± 0.01 | 300260 ± 550 | 1.57 ± 0.01 | 0.1777 ± 0.0008 |
| P1-4 | 70 | 109400 ± 330 | 1.39 ± 0.01 | 638120 ± 800 | 1.62 ± 0.01 | 0.1714 ± 0.0006 |
| P1-5 | 82 | 969810 ± 990 | 1.42 ± 0.01 | 4456550 ± 2120 | 1.67 ± 0.01 | 0.2176 ± 0.0002 |
| P1-6 | 98 | 856650 ± 930 | 1.39 ± 0.01 | 3861730 ± 1970 | 1.63 ± 0.01 | 0.2218 ± 0.0003 |
| P1-7 | 104 | 367490 ± 610 | 1.40 ± 0.01 | 1948330 ± 1400 | 1.73 ± 0.01 | 0.1886 ± 0.0003 |
| P1-8 | 19 | 66920 ± 260 | 1.45 ± 0.01 | 390600 ± 630 | 1.69 ± 0.01 | 0.1713 ± 0.0007 |
| P1-9 | 29 | 174670 ± 420 | 1.41 ± 0.01 | 850640 ± 930 | 1.65 ± 0.01 | 0.2053 ± 0.0005 |
| P1-10 | 42 | 319320 ± 570 | 1.33 ± 0.01 | 1767220 ± 1330 | 1.65 ± 0.01 | 0.1807 ± 0.0003 |
| P1-11 | 31 | 71910 ± 270 | 1.42 ± 0.01 | 427310 ± 660 | 1.59 ± 0.01 | 0.1683 ± 0.0007 |
| P1-12 | 38 | 63120 ± 250 | 1.43 ± 0.01 | 361350 ± 600 | 1.66 ± 0.01 | 0.1747 ± 0.0008 |
| P1-13 | 49 | 112230 ± 340 | 1.43 ± 0.01 | 562750 ± 750 | 1.65 ± 0.01 | 0.1994 ± 0.0007 |
| P1-14 | 61 | 868020 ± 940 | 1.42 ± 0.01 | 4274960 ± 2070 | 1.73 ± 0.01 | 0.2030 ± 0.0002 |
| ‍ | | | | | | |
| P2-1 | 13 | 30720 ± 180 | 1.36 ± 0.02 | 176120 ± 420 | 1.47 ± 0.01 | 0.1744 ± 0.0011 |
| P2-2 | 21 | 30470 ± 180 | 1.35 ± 0.02 | 194900 ± 440 | 1.54 ± 0.01 | 0.1563 ± 0.0010 |
| P2-3 | 27 | 30660 ± 180 | 1.36 ± 0.02 | 179200 ± 430 | 1.48 ± 0.01 | 0.1711 ± 0.0011 |
| P2-4 | 36 | 27270 ± 170 | 1.36 ± 0.02 | 201750 ± 450 | 1.37 ± 0.01 | 0.1352 ± 0.0009 |
| P2-5 | 44 | 17030 ± 140 | 1.33 ± 0.02 | 109750 ± 330 | 1.4 ± 0.01 | 0.1552 ± 0.0013 |
| P2-6 | 51 | 23380 ± 160 | 1.33 ± 0.02 | 150540 ± 390 | 1.43 ± 0.01 | 0.1553 ± 0.0011 |
| P2-7 | 59 | 16240 ± 130 | 1.37 ± 0.02 | 110340 ± 330 | 1.4 ± 0.01 | 0.1471 ± 0.0013 |
| P2-8 | 69 | 28090 ± 170 | 1.35 ± 0.02 | 167180 ± 410 | 1.44 ± 0.01 | 0.1680 ± 0.0011 |
| P2-9 | 73 | 35960 ± 190 | 1.44 ± 0.02 | 191070 ± 440 | 1.61 ± 0.01 | 0.1882 ± 0.0011 |
| P2-10 | 82 | 38160 ± 200 | 1.32 ± 0.01 | 310760 ± 560 | 1.63 ± 0.01 | 0.1228 ± 0.0007 |
| P2-11 | 90 | 178290 ± 430 | 1.39 ± 0.01 | 783240 ± 890 | 1.7 ± 0.01 | 0.2276 ± 0.0006 |
| P2-12 | 99 | 436900 ± 660 | 1.48 ± 0.01 | 1673510 ± 1300 | 1.79 ± 0.01 | 0.2611 ± 0.0004 |
| P2-13 | 108 | 558350 ± 750 | 1.43 ± 0.01 | 2314240 ± 1530 | 1.71 ± 0.01 | 0.2413 ± 0.0004 |
| P2-14 | 115 | 2068180 ± 1440 | 1.46 ± 0.01 | 7446540 ± 2740 | 1.84 ± 0.01 | 0.2777 ± 0.0002 |
| P2-15 | 123 | 1779180 ± 1340 | 1.44 ± 0.01 | 7234510 ± 2700 | 1.73 ± 0.01 | 0.2459 ± 0.0002 |
| P2-16 | 128 | 2497380 ± 1590 | 1.49 ± 0.01 | 9267760 ± 3050 | 1.85 ± 0.01 | 0.2695 ± 0.0002 |
| ‍ | | | | | | |
| P3-1 | 10 | - | - | - | - | - |
| P3-2 | 16 | 180040 ± 430 | 1.40 ± 0.01 | 774670 ± 880 | 1.6 ± 0.01 | 0.2324 ± 0.0006 |
| P3-3 | 24 | 245380 ± 500 | 1.43 ± 0.01 | 1012100 ± 1010 | 1.68 ± 0.01 | 0.2424 ± 0.0005 |
| P3-4 | 32 | 381170 ± 620 | 1.44 ± 0.01 | 1527940 ± 1240 | 1.7 ± 0.01 | 0.2495 ± 0.0005 |
| P3-5 | 38 | 278470 ± 530 | 1.38 ± 0.01 | 1219320 ± 1110 | 1.63 ± 0.01 | 0.2284 ± 0.0005 |
| P3-6 | 44 | 136350 ± 370 | 1.45 ± 0.01 | 605110 ± 780 | 1.72 ± 0.01 | 0.2253 ± 0.0007 |
| P3-7 | 52 | 119180 ± 350 | 1.42 ± 0.01 | 557280 ± 750 | 1.67 ± 0.01 | 0.2139 ± 0.0007 |
| P3-8 | 58 | 93780 ± 310 | 1.40 ± 0.01 | 479230 ± 700 | 1.54 ± 0.01 | 0.1957 ± 0.0007 |
| P3-9 | 196 | 3390720 ± 1850 | 1.45 ± 0.01 | 12624090 ± 3570 | 1.85 ± 0.01 | 0.2686 ± 0.0002 |
| P3-10 | 201 | 2980400 ± 1730 | 1.43 ± 0.01 | 12307810 ± 3520 | 1.83 ± 0.01 | 0.2422 ± 0.0002 |
| P3-11 | 206 | 2739760 ± 1660 | 1.44 ± 0.01 | 10511600 ± 3250 | 1.79 ± 0.01 | 0.2606 ± 0.0002 |
| P3-12 | 211 | 1703270 ± 1310 | 1.49 ± 0.01 | 5401880 ± 2330 | 1.73 ± 0.01 | 0.3153 ± 0.0003 |
| ‍ | | | | | | |
| P4-1 | 20 | - | - | - | - | - |
| P4-2 | 26 | 30170 ± 180 | 1.41 ± 0.02 | 163030 ± 410 | 1.6 ± 0.01 | 0.1850 ± 0.0012 |
| P4-3 | 32 | 23820 ± 160 | 1.34 ± 0.02 | 142360 ± 380 | 1.49 ± 0.01 | 0.1673 ± 0.0012 |
| P4-4 | 40 | 39220 ± 200 | 1.34 ± 0.01 | 217850 ± 470 | 1.52 ± 0.01 | 0.1800 ± 0.0010 |
| P4-5 | 46 | 56710 ± 240 | 1.42 ± 0.01 | 335470 ± 580 | 1.7 ± 0.01 | 0.1691 ± 0.0008 |
| P4-6 | 53 | 65290 ± 260 | 1.38 ± 0.01 | 339860 ± 590 | 1.49 ± 0.01 | 0.1921 ± 0.0008 |
| P4-7 | 59 | 67530 ± 260 | 1.35 ± 0.01 | 368140 ± 610 | 1.51 ± 0.01 | 0.1834 ± 0.0008 |
| P4-8 | 69 | 123410 ± 350 | 1.38 ± 0.01 | 559950 ± 750 | 1.6 ± 0.01 | 0.2204 ± 0.0007 |
| P4-9 | 72 | 158330 ± 400 | 1.42 ± 0.01 | 652120 ± 810 | 1.57 ± 0.01 | 0.2428 ± 0.0007 |
| P4-10 | 78 | 351070 ± 600 | 1.41 ± 0.01 | 1460540 ± 1210 | 1.7 ± 0.01 | 0.2404 ± 0.0005 |
| P4-11 | 86 | 438060 ± 670 | 1.39 ± 0.01 | 1798110 ± 1350 | 1.68 ± 0.01 | 0.2436 ± 0.0004 |
| P4-12 | 95 | 2330940 ± 1530 | 1.53 ± 0.01 | 7214910 ± 2700 | 1.89 ± 0.01 | 0.3231 ± 0.0002 |
| ‍ | | | | | | |
| P5-1 | 56 | 525450 ± 730 | 1.35 ± 0.01 | 2801640 ± 1680 | 1.61 ± 0.01 | 0.1876 ± 0.0003 |
| P5-2 | 62 | 576440 ± 760 | 1.42 ± 0.01 | 2538900 ± 1600 | 1.71 ± 0.01 | 0.2270 ± 0.0003 |
| P5-3 | 71 | 1149090 ± 1080 | 1.47 ± 0.01 | 4523200 ± 2130 | 1.81 ± 0.01 | 0.2540 ± 0.0003 |
| P5-4 | 78 | 761350 ± 880 | 1.42 ± 0.01 | 3238670 ± 1810 | 1.72 ± 0.01 | 0.2351 ± 0.0003 |
| P5-5 | 86 | 1899580 ± 1380 | 1.45 ± 0.01 | 6796840 ± 2620 | 1.8 ± 0.01 | 0.2795 ± 0.0002 |
| P5-6 | 93 | 2273440 ± 1510 | 1.44 ± 0.01 | 8754040 ± 2970 | 1.79 ± 0.01 | 0.2597 ± 0.0002 |
| P5-7 | 99 | 1363920 ± 1170 | 1.46 ± 0.01 | 5451870 ± 2340 | 1.85 ± 0.01 | 0.2502 ± 0.0002 |
| P5-8 | 106 | 1328730 ± 1160 | 1.45 ± 0.01 | 5174900 ± 2280 | 1.81 ± 0.01 | 0.2568 ± 0.0003 |
| P5-9 | 38 | 893760 ± 950 | 1.41 ± 0.01 | 3594520 ± 1900 | 1.73 ± 0.01 | 0.2486 ± 0.0003 |
| P5-10 | 59 | 731420 ± 860 | 1.38 ± 0.01 | 3359900 ± 1840 | 1.64 ± 0.01 | 0.2177 ± 0.0003 |
| P5-11 | 66 | 1305010 ± 1150 | 1.42 ± 0.01 | 5313050 ± 2310 | 1.76 ± 0.01 | 0.2456 ± 0.0002 |
| ‍ | | | | | | |
| P6-1 | 10 | 34510 ± 190 | 1.37 ± 0.02 | 228680 ± 480 | 1.44 ± 0.01 | 0.1509 ± 0.0009 |
| P6-2 | 17 | - | - | - | - | - |
| P6-3 | 24 | - | - | - | - | - |
| P6-4 | 32 | 98800 ± 320 | 1.37 ± 0.01 | 650630 ± 810 | 1.68 ± 0.01 | 0.1519 ± 0.0005 |
| P6-5 | 40 | 236440 ± 490 | 1.41 ± 0.01 | 1082050 ± 1040 | 1.68 ± 0.01 | 0.2185 ± 0.0005 |
| P6-6 | 52 | 326240 ± 570 | 1.43 ± 0.01 | 1450100 ± 1210 | 1.68 ± 0.01 | 0.2250 ± 0.0004 |
| P6-7 | 62 | 229420 ± 480 | 1.36 ± 0.01 | 1277870 ± 1140 | 1.58 ± 0.01 | 0.1795 ± 0.0004 |
| P6-8 | 69 | 145870 ± 390 | 1.42 ± 0.01 | 735940 ± 860 | 1.69 ± 0.01 | 0.1982 ± 0.0006 |
| P6-9 | 89 | 318260 ± 570 | 1.4 ± 0.01 | 1425850 ± 1200 | 1.64 ± 0.01 | 0.2232 ± 0.0004 |
| P6-10 | 94 | 797720 ± 900 | 1.46 ± 0.01 | 3153550 ± 1780 | 1.82 ± 0.01 | 0.2530 ± 0.0003 |
| P6-11 | 100 | 710680 ± 850 | 1.48 ± 0.01 | 2864650 ± 1700 | 1.88 ± 0.01 | 0.2481 ± 0.0003 |
| P6-12 | 109 | 598220 ± 780 | 1.44 ± 0.01 | 2534070 ± 1600 | 1.81 ± 0.01 | 0.2361 ± 0.0003 |
| ‍ | | | | | | |
| P7-1 | 11 | 22960 ± 160 | 1.37 ± 0.02 | 110760 ± 340 | 1.4 ± 0.01 | 0.2073 ± 0.0015 |
| P7-2 | 18 | 21360 ± 150 | 1.38 ± 0.02 | 126730 ± 360 | 1.49 ± 0.01 | 0.1686 ± 0.0013 |
| P7-3 | 27 | 8630 ± 100 | 1.38 ± 0.03 | 54430 ± 240 | 1.37 ± 0.01 | 0.1585 ± 0.0020 |
| P7-4 | 36 | 13470 ± 120 | 1.38 ± 0.02 | 72130 ± 270 | 1.45 ± 0.01 | 0.1867 ± 0.0018 |
| P7-5 | 45 | 18100 ± 140 | 1.43 ± 0.02 | 89730 ± 300 | 1.5 ± 0.01 | 0.2017 ± 0.0017 |
| P7-6 | 52 | 46250 ± 220 | 1.45 ± 0.01 | 191540 ± 440 | 1.59 ± 0.01 | 0.2415 ± 0.0013 |
| P7-7 | 61 | 212950 ± 460 | 1.51 ± 0.01 | 842500 ± 920 | 1.77 ± 0.01 | 0.2528 ± 0.0006 |
| P7-8 | 71 | 321620 ± 570 | 1.45 ± 0.01 | 1225730 ± 1110 | 1.72 ± 0.01 | 0.2624 ± 0.0005 |
| P7-9 | 83 | 264480 ± 520 | 1.41 ± 0.01 | 1123640 ± 1060 | 1.69 ± 0.01 | 0.2354 ± 0.0005 |
| P7-10 | 101 | 821220 ± 910 | 1.46 ± 0.01 | 2955720 ± 1730 | 1.78 ± 0.01 | 0.2778 ± 0.0003 |
| P7-11 | 206 | 262170 ± 520 | 1.42 ± 0.01 | 1109600 ± 1060 | 1.67 ± 0.01 | 0.2363 ± 0.0005 |
| P7-12 | 215 | 891300 ± 950 | 1.47 ± 0.01 | 2883660 ± 1700 | 1.75 ± 0.01 | 0.3091 ± 0.0004 |
| P7-13 | 223 | 1393890 ± 1190 | 1.48 ± 0.01 | 4607570 ± 2150 | 1.79 ± 0.01 | 0.3025 ± 0.0003 |
| P7-14 | 231 | 803950 ± 900 | 1.44 ± 0.01 | 2934380 ± 1720 | 1.68 ± 0.01 | 0.2740 ± 0.0003 |
| P7-15 | 237 | 1857250 ± 1370 | 1.46 ± 0.01 | 6484650 ± 2560 | 1.77 ± 0.01 | 0.2864 ± 0.0002 |
| P7-16 | 245 | 1421220 ± 1200 | 1.48 ± 0.01 | 5005670 ± 2250 | 1.84 ± 0.01 | 0.2839 ± 0.0003 |
| P7-17 | 251 | 1945160 ± 1400 | 1.43 ± 0.01 | 7893870 ± 2820 | 1.81 ± 0.01 | 0.2464 ± 0.0002 |
| P7-18 | 257 | 1723790 ± 1320 | 1.48 ± 0.01 | 6501760 ± 2560 | 1.86 ± 0.01 | 0.2651 ± 0.0002 |
| ‍ | | | | | | |
| P8-1 | 13 | 157010 ± 400 | 1.37 ± 0.01 | 829050 ± 910 | 1.59 ± 0.01 | 0.1894 ± 0.0005 |
| P8-2 | 21 | 313710 ± 560 | 1.40 ± 0.01 | 1414780 ± 1190 | 1.7 ± 0.01 | 0.2217 ± 0.0004 |
| P8-3 | 28 | 486680 ± 700 | 1.40 ± 0.01 | 2164140 ± 1480 | 1.67 ± 0.01 | 0.2249 ± 0.0004 |
| P8-4 | 35 | 831880 ± 920 | 1.41 ± 0.01 | 3599670 ± 1900 | 1.73 ± 0.01 | 0.2311 ± 0.0003 |
| P8-5 | 42 | 382270 ± 620 | 1.42 ± 0.01 | 1723570 ± 1320 | 1.74 ± 0.01 | 0.2218 ± 0.0004 |
| P8-6 | 50 | 202290 ± 450 | 1.42 ± 0.01 | 995450 ± 1000 | 1.7 ± 0.01 | 0.2032 ± 0.0005 |
| P8-7 | 57 | 111490 ± 340 | 1.43 ± 0.01 | 586800 ± 770 | 1.58 ± 0.01 | 0.1900 ± 0.0006 |
| P8-8 | 64 | 247340 ± 500 | 1.43 ± 0.01 | 1137310 ± 1070 | 1.72 ± 0.01 | 0.2175 ± 0.0005 |
| P8-9 | 72 | 594130 ± 770 | 1.42 ± 0.01 | 2585990 ± 1610 | 1.69 ± 0.01 | 0.2297 ± 0.0003 |
| P8-10 | 79 | 469550 ± 690 | 1.42 ± 0.01 | 2182170 ± 1480 | 1.74 ± 0.01 | 0.2152 ± 0.0003 |
| P8-11 | 85 | 918340 ± 960 | 1.42 ± 0.01 | 3774600 ± 1950 | 1.7 ± 0.01 | 0.2433 ± 0.0003 |
| P8-12 | 92 | - | - | - | - | - |
| P8-13 | 97 | 715140 ± 850 | 1.42 ± 0.01 | 3113870 ± 1770 | 1.71 ± 0.01 | 0.2297 ± 0.0003 |
| P8-14 | 102 | 410550 ± 640 | 1.42 ± 0.01 | 1929460 ± 1390 | 1.69 ± 0.01 | 0.2128 ± 0.0004 |
| P8-15 | 111 | 785250 ± 890 | 1.42 ± 0.01 | 3251570 ± 1810 | 1.7 ± 0.01 | 0.2415 ± 0.0003 |
| ‍ | | | | | | |
| P8a-1 | 26 | 105610 ± 330 | 1.39 ± 0.01 | 555640 ± 750 | 1.58 ± 0.01 | 0.1901 ± 0.0006 |
| P8a-2 | 36 | 377600 ± 620 | 1.43 ± 0.01 | 1909090 ± 1390 | 1.71 ± 0.01 | 0.1978 ± 0.0004 |
| P8a-3 | 46 | 532660 ± 730 | 1.4 ± 0.01 | 2751720 ± 1670 | 1.68 ± 0.01 | 0.1936 ± 0.0003 |
| P8a-4 | 56 | 380490 ± 620 | 1.41 ± 0.01 | 1766210 ± 1340 | 1.57 ± 0.01 | 0.2154 ± 0.0004 |
| P8a-5 | 64 | 105060 ± 330 | 1.42 ± 0.01 | 611710 ± 790 | 1.57 ± 0.01 | 0.1717 ± 0.0006 |
| P8a-6 | 72 | 396060 ± 630 | 1.47 ± 0.01 | 2022650 ± 1430 | 1.88 ± 0.01 | 0.1958 ± 0.0003 |
| P8a-7 | 81 | 305920 ± 560 | 1.45 ± 0.01 | 1414110 ± 1190 | 1.74 ± 0.01 | 0.2163 ± 0.0004 |
| P8a-8 | 89 | 460740 ± 680 | 1.43 ± 0.01 | 2539340 ± 1600 | 1.71 ± 0.01 | 0.1814 ± 0.0003 |
| P8a-9 | 96 | 1030050 ± 1020 | 1.46 ± 0.01 | 5203900 ± 2290 | 1.77 ± 0.01 | 0.1979 ± 0.0002 |
| P8a-10 | 108 | 890190 ± 950 | 1.49 ± 0.01 | 4151370 ± 2050 | 1.83 ± 0.01 | 0.2144 ± 0.0003 |
| ‍ | | | | | | |
| P9-1 | 16 | 13280 ± 120 | 1.32 ± 0.02 | 114370 ± 340 | 1.42 ± 0.01 | 0.1161 ± 0.0011 |
| P9-2 | 23 | 29420 ± 180 | 1.42 ± 0.02 | 175170 ± 420 | 1.52 ± 0.01 | 0.1679 ± 0.0011 |
| P9-3 | 28 | 49850 ± 230 | 1.37 ± 0.01 | 376150 ± 620 | 1.61 ± 0.01 | 0.1325 ± 0.0006 |
| P9-4 | 35 | 63690 ± 260 | 1.42 ± 0.01 | 360160 ± 600 | 1.53 ± 0.01 | 0.1768 ± 0.0008 |
| P9-5 | 41 | 133810 ± 370 | 1.41 ± 0.01 | 732590 ± 860 | 1.62 ± 0.01 | 0.1827 ± 0.0005 |
| P9-6 | 48 | 318030 ± 570 | 1.39 ± 0.01 | 1525510 ± 1240 | 1.59 ± 0.01 | 0.2085 ± 0.0004 |
| P9-7 | 55 | 123060 ± 350 | 1.37 ± 0.01 | 678710 ± 830 | 1.5 ± 0.01 | 0.1813 ± 0.0006 |
| P9-8 | 62 | 174670 ± 420 | 1.36 ± 0.01 | 893020 ± 950 | 1.55 ± 0.01 | 0.1956 ± 0.0005 |
| P9-9 | 71 | 108170 ± 330 | 1.36 ± 0.01 | 611160 ± 790 | 1.56 ± 0.01 | 0.1770 ± 0.0006 |
| P9-10 | 78 | 198610 ± 450 | 1.41 ± 0.01 | 1089700 ± 1050 | 1.69 ± 0.01 | 0.1823 ± 0.0004 |
| P9-11 | 95 | 242360 ± 500 | 1.41 ± 0.01 | 1239690 ± 1120 | 1.63 ± 0.01 | 0.1955 ± 0.0004 |
| P9-12 | 105 | 275350 ± 530 | 1.43 ± 0.01 | 1348320 ± 1170 | 1.66 ± 0.01 | 0.2042 ± 0.0004 |
| P9-13 | 117 | 200680 ± 450 | 1.39 ± 0.01 | 1133980 ± 1070 | 1.58 ± 0.01 | 0.1770 ± 0.0004 |
| P9-14 | 128 | 421670 ± 650 | 1.37 ± 0.01 | 2411350 ± 1560 | 1.67 ± 0.01 | 0.1749 ± 0.0003 |
| P9-15 | 146 | 413190 ± 650 | 1.4 ± 0.01 | 2417410 ± 1560 | 1.64 ± 0.01 | 0.1709 ± 0.0003 |
| ‍ | | | | | | |
| P10-1 | 17 | 532480 ± 730 | 1.42 ± 0.01 | 2093490 ± 1450 | 1.69 ± 0.01 | 0.2543 ± 0.0004 |
| P10-2 | 25 | 534350 ± 740 | 1.41 ± 0.01 | 2211090 ± 1490 | 1.84 ± 0.01 | 0.2417 ± 0.0004 |
| P10-3 | 33 | 606880 ± 780 | 1.41 ± 0.01 | 2385210 ± 1550 | 1.65 ± 0.01 | 0.2544 ± 0.0004 |
| P10-4 | 41 | 817460 ± 910 | 1.47 ± 0.01 | 2772060 ± 1670 | 1.73 ± 0.01 | 0.2949 ± 0.0004 |
| P10-5 | 50 | 1366970 ± 1170 | 1.47 ± 0.01 | 4384190 ± 2100 | 1.74 ± 0.01 | 0.3118 ± 0.0003 |
| P10-6 | 59 | 526360 ± 730 | 1.43 ± 0.01 | 1965900 ± 1410 | 1.67 ± 0.01 | 0.2677 ± 0.0004 |
| P10-7 | 66 | 206490 ± 460 | 1.47 ± 0.01 | 789350 ± 890 | 1.68 ± 0.01 | 0.2616 ± 0.0007 |
| P10-8 | 73 | 46460 ± 220 | 1.41 ± 0.01 | 213230 ± 460 | 1.53 ± 0.01 | 0.2179 ± 0.0011 |
| P10-9 | 81 | 465630 ± 690 | 1.45 ± 0.01 | 1729960 ± 1320 | 1.77 ± 0.01 | 0.2692 ± 0.0004 |
| P10-10 | 87 | 2297110 ± 1520 | 1.46 ± 0.01 | 7871980 ± 2820 | 1.81 ± 0.01 | 0.2918 ± 0.0002 |
| P10-11 | 95 | 2266730 ± 1510 | 1.47 ± 0.01 | 7300580 ± 2710 | 1.81 ± 0.01 | 0.3105 ± 0.0002 |
| P10-12 | 108 | 1046830 ± 1030 | 1.47 ± 0.01 | 3288240 ± 1820 | 1.78 ± 0.01 | 0.3184 ± 0.0004 |
| P10-23 | 26 | 186590 ± 440 | 1.50 ± 0.01 | 700990 ± 840 | 1.68 ± 0.01 | 0.2662 ± 0.0007 |
| P10-24 | 47 | 153880 ± 400 | 1.51 ± 0.01 | 625460 ± 790 | 1.8 ± 0.01 | 0.2460 ± 0.0007 |
| P10-25 | 66 | 90400 ± 300 | 1.44 ± 0.01 | 423110 ± 650 | 1.52 ± 0.01 | 0.2137 ± 0.0008 |
| ‍ | | | | | | |
| P11-1 | 41 | 126720 ± 360 | 1.44 ± 0.01 | 604760 ± 780 | 1.57 ± 0.01 | 0.2095 ± 0.0007 |
| P11-2 | 51 | 35580 ± 190 | 1.39 ± 0.02 | 196460 ± 450 | 1.45 ± 0.01 | 0.1811 ± 0.0011 |
| P11-3 | 62 | 28400 ± 170 | 1.36 ± 0.02 | 172120 ± 420 | 1.4 ± 0.01 | 0.1650 ± 0.0011 |
| P11-4 | 70 | 30180 ± 180 | 1.36 ± 0.02 | 205220 ± 460 | 1.4 ± 0.01 | 0.1471 ± 0.0009 |
| P11-5 | 77 | 24410 ± 160 | 1.39 ± 0.02 | 167660 ± 410 | 1.4 ± 0.01 | 0.1456 ± 0.0010 |
| P11-6 | 87 | 94690 ± 310 | 1.50 ± 0.01 | 410110 ± 640 | 1.68 ± 0.01 | 0.2309 ± 0.0008 |
| P11-7 | 96 | 56410 ± 240 | 1.34 ± 0.01 | 285900 ± 540 | 1.42 ± 0.01 | 0.1973 ± 0.0009 |
| P11-8 | 109 | 97650 ± 320 | 1.37 ± 0.01 | 514370 ± 720 | 1.48 ± 0.01 | 0.1898 ± 0.0007 |
| P11-9 | 116 | 76550 ± 280 | 1.35 ± 0.01 | 461200 ± 680 | 1.48 ± 0.01 | 0.166 ± 0.0007 |
| P11-10 | 125 | 88210 ± 300 | 1.35 ± 0.01 | 395350 ± 630 | 1.44 ± 0.01 | 0.2231 ± 0.0008 |
| P11-11 | 132 | 417990 ± 650 | 1.48 ± 0.01 | 1994910 ± 1420 | 1.79 ± 0.01 | 0.2095 ± 0.0004 |
| P11-12 | 141 | 160890 ± 410 | 1.47 ± 0.01 | 675510 ± 830 | 1.65 ± 0.01 | 0.2382 ± 0.0007 |
| P11-13 | 150 | 53090 ± 230 | 1.41 ± 0.01 | 281990 ± 530 | 1.47 ± 0.01 | 0.1883 ± 0.0009 |
| P11-14 | 158 | 262370 ± 520 | 1.39 ± 0.01 | 1063400 ± 1040 | 1.54 ± 0.01 | 0.2467 ± 0.0005 |
| P11-15 | 165 | 1582250 ± 1260 | 1.43 ± 0.01 | 6071660 ± 2470 | 1.63 ± 0.01 | 0.2606 ± 0.0002 |
| P11-16 | 173 | 1820950 ± 1360 | 1.42 ± 0.01 | 6794310 ± 2620 | 1.64 ± 0.01 | 0.268 ± 0.0002 |
| ‍ | | | | | | |
| P12-1 | 11 | - | - | - | - | - |
| P12-2 | 18 | 186170 ± 440 | 1.42 ± 0.01 | 774720 ± 880 | 1.65 ± 0.01 | 0.2403 ± 0.0006 |
| P12-3 | 27 | 120110 ± 350 | 1.41 ± 0.01 | 555750 ± 750 | 1.64 ± 0.01 | 0.2161 ± 0.0007 |
| P12-4 | 37 | 127670 ± 360 | 1.42 ± 0.01 | 527090 ± 730 | 1.59 ± 0.01 | 0.2422 ± 0.0008 |
| P12-5 | 48 | 91340 ± 310 | 1.35 ± 0.01 | 439940 ± 670 | 1.58 ± 0.01 | 0.2076 ± 0.0008 |
| P12-6 | 59 | 104890 ± 330 | 1.39 ± 0.01 | 453260 ± 680 | 1.55 ± 0.01 | 0.2314 ± 0.0008 |
| P12-7 | 67 | 108490 ± 330 | 1.35 ± 0.01 | 516530 ± 720 | 1.52 ± 0.01 | 0.2100 ± 0.0007 |
| P12-8 | 76 | 160250 ± 400 | 1.41 ± 0.01 | 687280 ± 830 | 1.61 ± 0.01 | 0.2332 ± 0.0007 |
| P12-9 | 84 | 334660 ± 580 | 1.40 ± 0.01 | 1261190 ± 1130 | 1.63 ± 0.01 | 0.2653 ± 0.0005 |
| P12-10 | 94 | 500860 ± 710 | 1.38 ± 0.01 | 1992620 ± 1420 | 1.62 ± 0.01 | 0.2514 ± 0.0004 |
| P12-11 | 100 | 1030140 ± 1020 | 1.42 ± 0.01 | 3989850 ± 2000 | 1.76 ± 0.01 | 0.2582 ± 0.0003 |
| P12-12 | 108 | 2090650 ± 1450 | 1.41 ± 0.01 | 7844140 ± 2810 | 1.71 ± 0.01 | 0.2665 ± 0.0002 |
| P12-13 | 48 | 613700 ± 790 | 1.41 ± 0.01 | 2178620 ± 1480 | 1.63 ± 0.01 | 0.2817 ± 0.0004 |
| P12-14 | 57 | 204340 ± 460 | 1.37 ± 0.01 | 939300 ± 970 | 1.55 ± 0.01 | 0.2175 ± 0.0005 |
| P12-15 | 67 | 546250 ± 740 | 1.35 ± 0.01 | 2292340 ± 1520 | 1.55 ± 0.01 | 0.2383 ± 0.0004 |
| P12-16 | 76 | 1473540 ± 1220 | 1.48 ± 0.01 | 5242030 ± 2300 | 1.79 ± 0.01 | 0.2811 ± 0.0003 |
| P12-17 | 82 | 1182320 ± 1090 | 1.48 ± 0.01 | 4119750 ± 2040 | 1.8 ± 0.01 | 0.287 ± 0.0003 |
| ‍ | | | | | | |
| P13-1 | 12 | 393530 ± 630 | 1.45 ± 0.01 | 1518620 ± 1240 | 1.62 ± 0.01 | 0.2591 ± 0.0005 |
| P13-2 | 19 | 997920 ± 1000 | 1.50 ± 0.01 | 3491880 ± 1870 | 1.81 ± 0.01 | 0.2858 ± 0.0003 |
| P13-3 | 28 | 767060 ± 880 | 1.46 ± 0.01 | 2737770 ± 1660 | 1.69 ± 0.01 | 0.2802 ± 0.0004 |
| P13-4 | 35 | 1482330 ± 1220 | 1.44 ± 0.01 | 5747930 ± 2410 | 1.73 ± 0.01 | 0.2579 ± 0.0002 |
| P13-5 | 53 | 1663480 ± 1300 | 1.44 ± 0.01 | 5823600 ± 2420 | 1.71 ± 0.01 | 0.2856 ± 0.0003 |
| P13-6 | 90 | 2179850 ± 1480 | 1.45 ± 0.01 | 7616640 ± 2770 | 1.74 ± 0.01 | 0.2862 ± 0.0002 |
| P13-7 | 119 | 2578670 ± 1610 | 1.46 ± 0.01 | 9922150 ± 3160 | 1.88 ± 0.01 | 0.2599 ± 0.0002 |
| P13-8 | 129 | 239610 ± 490 | 1.42 ± 0.01 | 956790 ± 980 | 1.62 ± 0.01 | 0.2504 ± 0.0006 |
| P13-9 | 95 | 1019400 ± 1010 | 1.43 ± 0.01 | 3950050 ± 1990 | 1.76 ± 0.01 | 0.2581 ± 0.0003 |
| P13-10 | 94 | 852760 ± 930 | 1.45 ± 0.01 | 3134130 ± 1780 | 1.71 ± 0.01 | 0.2721 ± 0.0003 |
| ‍ | | | | | | |
| P14-1 | 19 | 131500 ± 370 | 1.36 ± 0.01 | 633820 ± 800 | 1.5 ± 0.01 | 0.2075 ± 0.0006 |
| P14-2 | 30 | 291300 ± 540 | 1.41 ± 0.01 | 1338610 ± 1160 | 1.7 ± 0.01 | 0.2176 ± 0.0004 |
| P14-3 | 41 | 351300 ± 600 | 1.43 ± 0.01 | 1420180 ± 1200 | 1.65 ± 0.01 | 0.2474 ± 0.0005 |
| P14-4 | 53 | 589520 ± 770 | 1.42 ± 0.01 | 2312780 ± 1530 | 1.69 ± 0.01 | 0.2549 ± 0.0004 |
| P14-5 | 64 | 1058100 ± 1030 | 1.40 ± 0.01 | 4335030 ± 2090 | 1.7 ± 0.01 | 0.2441 ± 0.0003 |
| P14-6 | 75 | 1074900 ± 1040 | 1.41 ± 0.01 | 4236450 ± 2070 | 1.65 ± 0.01 | 0.2537 ± 0.0003 |
| P14-7 | 87 | 1238450 ± 1120 | 1.44 ± 0.01 | 4420550 ± 2110 | 1.76 ± 0.01 | 0.2802 ± 0.0003 |
| P14-8 | 96 | 2536090 ± 1600 | 1.43 ± 0.01 | 9071720 ± 3020 | 1.86 ± 0.01 | 0.2796 ± 0.0002 |
| P14-9 | 80 | 1217600 ± 1110 | 1.46 ± 0.01 | 4310910 ± 2080 | 1.78 ± 0.01 | 0.2824 ± 0.0003 |
| P14-10 | 90 | 2323610 ± 1530 | 1.50 ± 0.01 | 7168170 ± 2690 | 1.79 ± 0.01 | 0.3242 ± 0.0002 |
| P14-11 | 61 | 817910 ± 910 | 1.42 ± 0.01 | 3016540 ± 1740 | 1.68 ± 0.01 | 0.2711 ± 0.0003 |
| P14-12 | 79 | 1895360 ± 1380 | 1.47 ± 0.01 | 6276030 ± 2510 | 1.79 ± 0.01 | 0.3020 ± 0.0003 |
| ‍ | | | | | | |
| P15-1 | 9 | 205610 ± 460 | 1.40 ± 0.01 | 918720 ± 960 | 1.55 ± 0.01 | 0.2238 ± 0.0005 |
| P15-2 | 16 | 243010 ± 500 | 1.39 ± 0.01 | 1131650 ± 1070 | 1.58 ± 0.01 | 0.2147 ± 0.0005 |
| P15-3 | 22 | 279100 ± 530 | 1.44 ± 0.01 | 1261740 ± 1130 | 1.66 ± 0.01 | 0.2212 ± 0.0005 |
| P15-4 | 29 | 408780 ± 640 | 1.41 ± 0.01 | 1756040 ± 1330 | 1.57 ± 0.01 | 0.2328 ± 0.0004 |
| P15-5 | 37 | 390880 ± 630 | 1.36 ± 0.01 | 1769870 ± 1340 | 1.61 ± 0.01 | 0.2209 ± 0.0004 |
| P15-6 | 52 | 351450 ± 600 | 1.37 ± 0.01 | 1573490 ± 1260 | 1.53 ± 0.01 | 0.2234 ± 0.0004 |
| P15-7 | 83 | 2759380 ± 1670 | 1.48 ± 0.01 | 9555070 ± 3100 | 1.78 ± 0.01 | 0.2888 ± 0.0002 |
| P15-8 | 109 | 326460 ± 580 | 1.42 ± 0.01 | 1283760 ± 1140 | 1.62 ± 0.01 | 0.2543 ± 0.0005 |
| P15-9 | 121 | 913830 ± 960 | 1.42 ± 0.01 | 3742090 ± 1940 | 1.68 ± 0.01 | 0.2442 ± 0.0003 |
| P15-10 | 137 | 1636030 ± 1280 | 1.49 ± 0.01 | 5667480 ± 2390 | 1.78 ± 0.01 | 0.2887 ± 0.0003 |
| P15-11 | 86 | 953120 ± 980 | 1.47 ± 0.01 | 3385680 ± 1850 | 1.74 ± 0.01 | 0.2815 ± 0.0003 |
| P15-12 | 94 | 939580 ± 970 | 1.44 ± 0.01 | 3304320 ± 1820 | 1.67 ± 0.01 | 0.2843 ± 0.0003 |
| ‍ | | | | | | |
| P16-1 | 9 | 94240 ± 310 | 1.35 ± 0.01 | 527740 ± 730 | 1.52 ± 0.01 | 0.1786 ± 0.0006 |
| P16-2 | 15 | 172740 ± 420 | 1.41 ± 0.01 | 816260 ± 910 | 1.54 ± 0.01 | 0.2116 ± 0.0006 |
| P16-3 | 22 | 458630 ± 680 | 1.4 ± 0.01 | 1911800 ± 1390 | 1.65 ± 0.01 | 0.2399 ± 0.0004 |
| P16-4 | 27 | 554980 ± 750 | 1.41 ± 0.01 | 2436910 ± 1570 | 1.64 ± 0.01 | 0.2277 ± 0.0003 |
| P16-5 | 33 | 614300 ± 790 | 1.41 ± 0.01 | 2659160 ± 1640 | 1.67 ± 0.01 | 0.2310 ± 0.0003 |
| P16-6 | 58 | 418030 ± 650 | 1.43 ± 0.01 | 1717840 ± 1320 | 1.64 ± 0.01 | 0.2433 ± 0.0004 |
| ‍ | | | | | | |
| P17-1 | 16 | 229860 ± 480 | 1.36 ± 0.01 | 1252790 ± 1120 | 1.59 ± 0.01 | 0.1835 ± 0.0004 |
| P17-2 | 27 | 833830 ± 920 | 1.46 ± 0.01 | 3626950 ± 1910 | 1.78 ± 0.01 | 0.2299 ± 0.0003 |
| P17-3 | 37 | 862830 ± 930 | 1.41 ± 0.01 | 4174970 ± 2050 | 1.75 ± 0.01 | 0.2067 ± 0.0002 |
| P17-4 | 45 | 685820 ± 830 | 1.44 ± 0.01 | 3580350 ± 1900 | 1.74 ± 0.01 | 0.1916 ± 0.0003 |
| P17-5 | 52 | 1204750 ± 1100 | 1.43 ± 0.01 | 5514220 ± 2360 | 1.73 ± 0.01 | 0.2185 ± 0.0002 |
| P17-6 | 60 | 471800 ± 690 | 1.4 ± 0.01 | 2243780 ± 1500 | 1.68 ± 0.01 | 0.2103 ± 0.0003 |
| P17-7 | 66 | 312940 ± 560 | 1.41 ± 0.01 | 1408690 ± 1190 | 1.65 ± 0.01 | 0.2222 ± 0.0004 |
| P17-8 | 73 | 1005820 ± 1010 | 1.4 ± 0.01 | 4855580 ± 2210 | 1.67 ± 0.01 | 0.2071 ± 0.0002 |
| P17-9 | 79 | 867040 ± 940 | 1.45 ± 0.01 | 3881110 ± 1980 | 1.78 ± 0.01 | 0.2234 ± 0.0003 |
| P17-10 | 80 | 657470 ± 820 | 1.44 ± 0.01 | 2954450 ± 1730 | 1.73 ± 0.01 | 0.2225 ± 0.0003 |
